# Integrated multi-omics investigation revealed the importance of phenylpropanoid metabolism in the defense response of *Lilium regale* Wilson to fusarium wilt

**DOI:** 10.1093/hr/uhae140

**Published:** 2024-05-20

**Authors:** Jie Deng, Xiaoli Che, Yue Gu, Yuan Qu, Diqiu Liu

## Abstract

Lilies (genus *Lilium*) play a significant role in the global cut-flower industry, but they are highly susceptible to fusarium wilt caused by *Fusarium oxysporum*. However, *Lilium regale*, a wild lily species, exhibits remarkable resistance to *F. oxysporum*. To investigate the quantitative resistance of *L. regale* to fusarium wilt, a comprehensive multi-omics analysis was conducted. Upon inoculation with *F. oxysporum*, *L. regale* roots showed a significant accumulation of phenylpropane metabolites, including lignin precursors, flavonoids, and hydroxycinnamic acids. These findings were consistent with the upregulated expression of phenylpropanoid biosynthesis-related genes encoding various enzymes, as revealed by transcriptomics and proteomics analyses. Furthermore, metabolomics and proteomics data demonstrated differential activation of monoterpenoid and isoquinoline alkaloid biosynthesis. Colorimetry and high-performance liquid chromatography analyses revealed significantly higher levels of total flavonoids, lignin, ferulic acid, phlorizin, and quercetin contents in *L. regale* scales compared with susceptible lily ‘Siberia’ scales during *F. oxysporum* infection. These phenylpropanes exhibited inhibitory effects on *F. oxysporum* growth and suppressed the expression of pathogenicity-related genes. Transcriptional regulatory network analysis suggested that ethylene-responsive transcription factors (ERFs) may positively regulate phenylpropanoid biosynthesis. Therefore, *LrERF4* was cloned and transiently overexpressed in the fusarium wilt-susceptible Oriental hybrid lily ‘Siberia’. The overexpression of *LrERF4* resulted in increased levels of total flavonoids, lignin, ferulic acid, phlorizin, and quercetin, while the silencing of *LrERF4* in *L. regale* through RNAi had the opposite effect. In conclusion, phenylpropanoid metabolism plays a crucial role in the defense response of *L. regale* against fusarium wilt, with *LrERF4* acting as a positive regulator of phenylpropane biosynthesis.

## Introduction

Plants face numerous stresses throughout their life cycle, including fungal diseases that have a detrimental impact on their growth and productivity. Over time, plants have developed unique immune mechanisms to combat pathogens [1]. Plant pattern recognition receptors detect pathogen-associated molecular patterns, triggering pattern-triggered immunity, which is closely linked to quantitative resistance (QTR) [2]. Pathogens release effector proteins to infect plants, but plants produce resistance proteins encoded by resistance genes to counteract these effectors, resulting in effector-triggered immunity, also known as single-gene resistance or qualitative resistance [3]. QTR, controlled by multiple genes and involving the biosynthesis of resistance-related metabolites, is considered the superior disease resistance mechanism in plants [4, 5].

Recent multi-omics analyses have provided comprehensive insights into the mechanisms underlying QTR. Metabolome and transcriptome analyses of *Zea mays* infected with *Puccinia sorghi* demonstrated higher accumulation of phenylpropanes and terpenes in the resistant line (H95:Rp1-D) compared with the susceptible line (H95) [6]. Additionally, transcription factors (TFs) such as WRKY, MYB, and bHLH (basic helix–loop–helix) were identified as regulators of the H95:Rp1-D response to *P. sorghi* infection. Metabolome and proteome analyses of *Triticum aestivum* indicated higher accumulation of hydroxycinnamic acid amides and flavonoids in the *Fusarium graminearum*-resistant wheat line HC374, leading to a thickened cell wall and enhanced resistance to *F. graminearum* [7]. Furthermore, integrated transcriptomic and metabolomic analyses revealed that the *Citrus sinensis* mutant MT exhibited increased resistance to *Penicillium digitatum* compared with the wild-type *C. sinensis* due to enhanced jasmonate biosynthesis [8].

Metabolites play a crucial role in plant defense responses, with several phenylpropanes derived from phenylalanine, terpenoids derived from methyl-glutaric acid, and alkaloids serving as secondary metabolites contributing to stress resistance [9]. For example, cinnamaldehyde, a phenylpropane, inhibits the growth of *Fusarium sambucinum* [10]. The β-costic acid in *Z. mays* restricts the growth of *Fusarium verticillioides* and *F. graminearum* both *in vivo* and *in vitro* [11]. Harmane, a β-carboline alkaloid extracted from *Peganum harmala*, damages the cell membrane of *Fusarium oxysporum* and induces its death [12]. Previous research has demonstrated that identifying and characterizing resistance-related genes using forward genetics-based methods, along with analyses of resistance-related metabolites, can elucidate the molecular basis of QTR [13].

Some TFs, including WRKY, MYB, bHLH, and ethylene-responsive TFs (ERFs), have been reported to modulate metabolite biosynthesis in plant immune systems [14]. ERFs, which belong to the AP2/ERF superfamily, can be further classified into ERF and CBF/DREB subfamilies based on the *cis*-acting elements they bind to (AGCCGCC and A/GCCGAC, respectively) [15]. In *Glycine max*, MAPK4/MAPK6 phosphorylates GmERF113, enhancing the expression of *GmPR1* and increasing resistance to *Phytophthora sojae* [16]. *Malus domestica* ERF114 positively regulates resistance to *Fusarium solani* by modulating the expression of *MdPRX63* (peroxidase 63) and enhancing lignin biosynthesis [17]. *Nicotiana benthamiana* ERF-IX-33 binds to the *NbEAS4* promoter, leading to increased phytoalexin (capsidiol) biosynthesis and improved resistance to *Phytophthora infestans* [18]. In *Arabidopsis thaliana*, ERF72 phosphorylated by AtMPK3/AtMPK6 promotes the accumulation of camalexin, thereby increasing resistance to *Botrytis cinerea* [19].

Lilies (*Lilium* spp.) are highly valued cut-flower species worldwide, but many lilies are susceptible to fusarium wilt, primarily caused by *F. oxysporum*, which negatively impacts lily production [20]. *Fusarium oxysporum* infection leads to root and scale decay, vascular bundle blockage, and hindered nutrient transport, ultimately resulting in wilting and death [21]. Fortunately, some wild lilies, including *Lilium regale* Wilson, exhibit resistance to fusarium wilt. Previous studies have shown that WRKY TFs in *L. regale* modulate the expression of resistance genes, such as *pathogenesis-related protein 10* (*PR10*), *chitinase*, and *defensin*, positively regulating resistance to *F. oxysporum* [22–24]. However, the mechanism underlying *L. regale*’s resistance to *F. oxysporum* remains unknown. This study aims to explore the metabolites associated with fusarium wilt resistance in *L. regale* using multi-omics techniques and investigate the related TFs as well as the antifungal effects of these metabolites.

## Results

### Transcriptome profile of *L. regale* responding to an *F. oxysporum* infection

**Figure 1:**
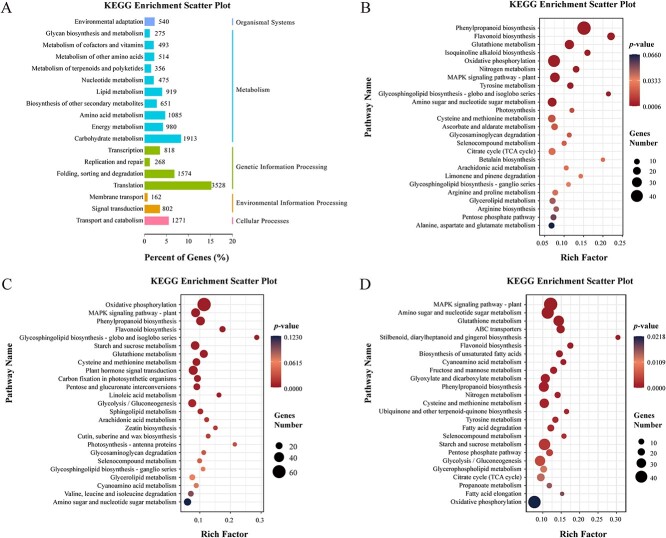
Transcriptome analysis of *L. regale* roots after inoculation with *F. oxysporum*. **A** KEGG enrichment analysis of total unigenes. **B**, **C**, **D** KEGG enrichment of differentially expressed genes at 2, 4, and 6 dpi, respectively. Bubble size reflects the number of genes annotated in a specific KEGG pathway, and color signifies the *P*-value.

Kyoto Encyclopedia of Genes and Genomes (KEGG) enrichment analysis revealed that 7661 unigenes in the *L. regale* transcriptomic data were associated with metabolism, with a significant number of unigenes involved in secondary metabolite biosynthesis (Fig. 1A). Consequently, a substantial number of metabolism-related unigenes were further identified.

In comparison with the control group (mock inoculation with sterile water), the expression of 752, 1150, and 4297 unigenes was upregulated at 2, 4, and 6 days post-inoculation (dpi), respectively, in the *F. oxysporum*-infected samples. Conversely, 2060, 2195, and 1224 unigenes exhibited downregulated expression levels at 2, 4, and 6 dpi, respectively, in the *F. oxysporum*-infected samples. KEGG pathway enrichment analysis of the metabolism-related differentially expressed genes (DEGs) indicated that the DEGs at 2 dpi were primarily associated with phenylpropanoid, flavonoid, and isoquinoline alkaloid biosynthesis, glutathione metabolism, and oxidative phosphorylation (Fig. 1B and Dynamic Fig. 1). At 4 dpi, many DEGs were related to mitogen-activated protein kinase (MAPK) signaling, oxidative phosphorylation, and phenylpropanoid biosynthesis (Fig. 1C). At 6 dpi, numerous DEGs were relevant to MAPK signaling, ABC transporters, glutathione metabolism, and stilbene/phenylpropanoid/diarylheptanoid/gingerol biosynthesis (Fig. 1D). The phenylpropanoid biosynthesis pathway serves as an upstream pathway for the synthesis of lignin/lignan, flavonoids, and coumarin. Moreover, the MAPK signaling pathway plays a role in regulating biotic and abiotic stress responses through interactions with plant hormone signaling pathways and TFs [25]. Additionally, downregulated oxidative phosphorylation, indicative of inhibited energy metabolism, may contribute to maintaining a balance between growth and immune responses. Therefore, the transcriptome analysis uncovered the induction of biosynthesis pathways for resistance-related metabolites and signaling pathways in *F. oxysporum*-infected *L. regale*.

### Characteristics of the proteome of *L. regale* inoculated with *F. oxysporum*

The *L. regale* proteins affected by *F. oxysporum* infection were identified using ultra-high-performance liquid chromatography–tandem mass spectrometry (UPLC–MS/MS). Out of the 473 595 secondary spectra obtained, 51 691 matched the spectra of known proteins. Additionally, 27 558 peptides were identified, including 26 053 unique peptides. A total of 6501 proteins were assembled and quantified based on the proteome data analysis. The differentially expressed proteins (DEPs) identified underwent KEGG pathway enrichment analysis. At 2, 4, and 6 dpi, 595, 652, and 657 upregulated DEPs were observed, respectively, while 398, 353, and 450 DEPs were downregulated, respectively.

**Figure 2:**
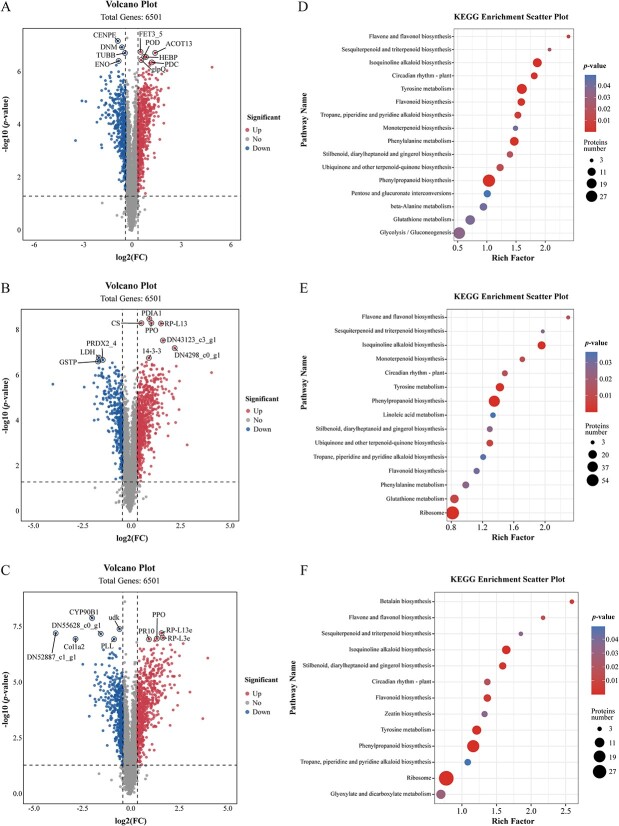
Proteome analysis of *L. regale* roots after inoculation with *F. oxysporum*. **A**, **B**, **C** Volcano plots of DEPs at 2, 4, and 6 dpi, respectively. Proteins with up- and downregulated expression profiles are marked with red and blue dots, respectively. **D**, **E**, **F** KEGG enrichment analysis of differentially expressed proteins at 2, 4, and 6 dpi, respectively. Bubble size reflects the number of genes annotated in a specific KEGG pathway, and color signifies the *P*-value.

Significantly, the antioxidant enzyme peroxidase (POD) and the energy metabolism-related enzymes pyruvate decarboxylase (PDC) and glycerophosphoryl diester phosphodiesterase (glpQ) showed increased abundance (Fig. 2A). At 4 dpi, the 14-3-3 protein, the antioxidant enzyme polyphenol oxidase (PPO), and the large subunit ribosomal protein L13e (RPL-13e) were upregulated (Fig. 2B). At 6 dpi, there was a significant increase in PPO, PR10, and RPL-13e, while the abundance of steroid 22*S*-hydroxylase (CYP90B1), a key enzyme involved in brassinosteroid biosynthesis, decreased (Fig. 2C). Thus, proteins with antifungal and antioxidant properties, such as pathogenesis-related proteins and antioxidant proteins (e.g. POD), were responsive to *F. oxysporum* infection.

The KEGG analysis revealed that at 2 dpi the DEPs were primarily associated with 16 KEGG pathways (*P* < 0.05) (Fig. 2D and Dynamic Fig. 2). Specifically, 33, 19, and 14 DEPs were related to phenylpropanoid biosynthesis, tyrosine metabolism, and isoquinoline alkaloid biosynthesis, respectively. Among the DEPs at 4 dpi, 15 KEGG pathways were enriched (Fig. 2E). Notably, 68, 44, and 18 DEPs were associated with the ribosome, phenylpropanoid biosynthesis, and tyrosine metabolism, respectively. At 6 dpi, the DEPs were involved in 13 KEGG pathways (Fig. 2F), with 72, 42, and 17 DEPs related to the ribosome, phenylpropanoid biosynthesis, and tyrosine metabolism, respectively. Interestingly, secondary metabolism-related pathways, including phenylpropanoid and isoquinoline alkaloid biosynthesis, were differentially activated at the three sampling time points following *F. oxysporum* inoculation.

### Overview of the metabolome of *L. regale* infected with *F. oxysporum*

**Figure 3:**
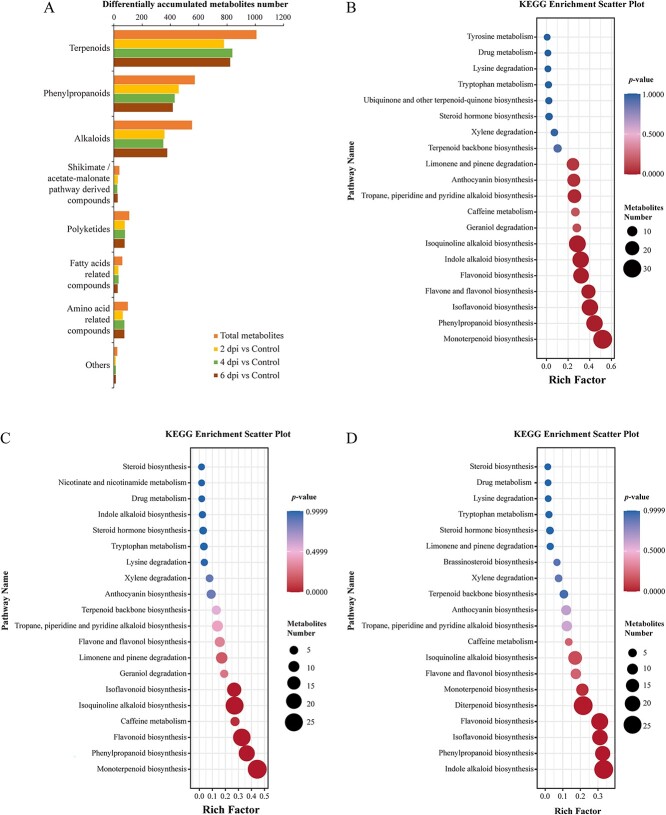
Metabolome analysis of *L. regale* roots after inoculation with *F. oxysporum*. **A** KEGG phytochemical compound classification of DAMs. **B**, **C**, **D** KEGG enrichment analysis of differentially accumulated metabolites at 2, 4, and 6 dpi, respectively. Bubble size reflects the number of genes annotated in a specific KEGG pathway, and color signifies the *P*-value.

The metabolome of *F. oxysporum*-inoculated *L. regale* was analyzed using UPLC–MS/MS, which detected 22 387 negative ions and 20 050 positive ions. A total of 15 341 ions were annotated as different metabolites in the KEGG database, with 12 172 ions representing phytochemical compounds, including 2460 ions corresponding to secondary metabolites. Among these, 1909 secondary metabolites showed significant differences in accumulation levels and were designated as differentially accumulated metabolites (DAMs). At 2, 4, and 6 dpi, 1340, 1115, and 1071 DAMs were upregulated, while 569, 794, and 838 DAMs were downregulated, respectively. The DAMs were classified into eight major categories according to the KEGG database (Fig. 3A). Terpenoids, phenylpropanes, and alkaloids represented the three major secondary metabolite groups that exhibited the most significant differential accumulation in *L. regale*, with 1007, 572, and 552 compounds classified as such, respectively. Notably, 80% of the identified phenylpropanes showed differential accumulation between the inoculated and control samples at 2 dpi, highlighting their importance during the early stages of *F. oxysporum* infection. Furthermore, 83 and 82% of the identified terpenoids showed differential accumulation between the inoculated and control samples at 4 and 6 dpi, respectively.

The KEGG enrichment analysis (Fig. 3B–D and Dynamic Fig. 3) revealed that the DAMs at 2 dpi were associated with monoterpenoid, phenylpropanoid, and isoflavonoid biosynthesis (Fig. 3B). At 4 dpi, 27, 24, and 21 DAMs were linked to monoterpenoid, phenylpropanoid, and flavonoid biosynthesis, respectively (Fig. 3C). The DAMs at 6 dpi were related to indole alkaloid, phenylpropanoid, and isoflavonoid biosynthesis (Fig. 3D). Additionally, primary metabolic pathways such as lysine degradation and tryptophan metabolism showed significant differential expression at 2, 4, and 6 dpi. Other differentially activated pathways included caffeine metabolism, limonene and pinene degradation, and steroid hormone biosynthesis. Phenylpropanes, including isoflavonoids, flavonoids, and flavonols, exhibited clear induction at all three time points following *F. oxysporum* inoculation. Monoterpenoids and isoquinoline alkaloids were also induced at all three time points.

### Integrated analysis of multi-omics data revealed the importance of phenylpropanes for *L. regale* resistance to *F. oxysporum*

**Figure 4:**
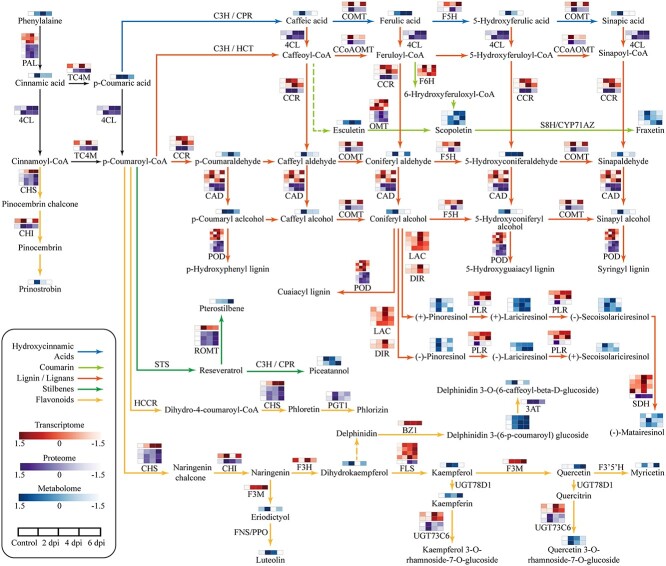
Multi-osmic integrative analysis of phenylpropanoid biosynthesis in *L. regale* during *F. oxysporum* infection. The black line represents a common pathway for phenylpropanoid biosynthesis. The blue line represents synthesis of hydroxycinnamic acids. The light green line represents coumarin biosynthesis. The red line represents lignin/lignan biosynthesis. The dark green line represents stilbene biosynthesis. The yellow line represents flavonoid biosynthesis. The full names of catalytic enzymes can be found in Supplementary Data Table S1.

The integrated analysis of transcriptome, proteome, and metabolome data revealed that the phenylpropanoid biosynthesis pathway was highly upregulated in *F. oxysporum*-infected *L. regale* roots (Fig. 4). The accumulation of hydroxycinnamic acids, such as ferulic acid and *p*-coumaric acid, increased at 2 dpi. Notably, the ferulic acid content increased 2.5-fold at 2 dpi. Additionally, the biosynthesis of lignin/lignans was also activated, as evidenced by the 3.14-, 2.44-, and 1.38-fold increases in the accumulation of (+)-lariciresinol, (+)-pinoresinol, and secoisolariciresinol at 2, 4, and 2 dpi, respectively. The lignin biosynthesis precursors were also upregulated. The biosynthesis of coumarins, including esculetin, scopoletin, and fraxetin, was induced, too. Similarly, the flavonoid contents (e.g. luteolin, kaempferol, quercetin, and pinostrobin) also increased.

We further analyzed the effects of the *F. oxysporum* infection on the expression levels of genes and the accumulation of proteins related to phenylpropanoid biosynthesis. Key enzymes involved in phenylpropanoid biosynthesis, such as phenylalanine ammonia lyase (PAL), showed significant increases at the transcriptome and proteome levels. The hydroxycinnamic acid synthesis-related enzyme caffeic acid 3-*O*-methyltransferase (COMT) was upregulated at 2 dpi , with 2.84- and 1.72-fold increase (relative to the corresponding control levels) at transcription and protein level, respectively. Moreover, the unigene and protein of ferulic acid 5-hydroxylase (F5H), another hydroxycinnamic acid synthesis-related enzyme, was upregulated at 4 dpi with 4.6- and 1.3-fold increase (relative to the corresponding control levels), respectively. Additionally, the production of cinnamoyl-CoA reductase (CCR), POD, dirigent (DIR), and laccase (LAC) was also upregulated at the transcriptome and proteome levels. Furthermore, the first enzyme of the flavonoid biosynthesis pathway, chalcone synthase (CHS), was significantly upregulated according to the transcriptome and proteome. Moreover, the expression levels of chalcone isomerase (CHI), flavonoid 3′-monooxygenase (F3M) and phlorizin synthase (PGT1), which affect flavonoid biosynthesis, increased at the transcript and protein levels during the infection of *L. regale* by *F. oxysporum*.

The multi-omics analysis also detected upregulated expression of terpenoid/alkaloid biosynthesis after inoculation with *F. oxysporum* (Supplementary Data Figs S1 and S2). The contents of some alkaloids, including 17-*O*-acetylnorajmaline, 17-*O*-acetylajmaline, norajmaline, 1,2-dihydrovomilenine, and ajmaline, increased during *F. oxysporum* infection. The expression of the upstream gene of ajmaline biosynthesis, *acetylajmaline esterase*, was upregulated at 2, 4, and 6 dpi (Supplementary Data Fig. S1). Similarly, the analysis of metabolomics and transcriptomics data indicated that dopamine biosynthesis was induced by *F. oxysporum*; moreover, terpenoid biosynthesis was influenced by *F. oxysporum* infection (Supplementary Data Fig. S2). In addition, expression of genes related to gibberellin biosynthesis was upregulated in *F. oxysporum-*infected *L. regale* roots (Supplementary Data Fig. S2).

Overall, the integrated analysis of multi-omics data highlighted the significant accumulation of phenylpropanes in *F. oxysporum*-infected *L. regale*, indicating their importance in the defense response against the pathogen. Furthermore, the biosynthesis pathways of alkaloids and terpenoids were significantly affected by the infection. These findings provide valuable insights into the molecular mechanisms underlying the defense response.

### Phenylpropanes accumulated more in the resistant *L. regale* than in the susceptible lily ‘Siberia’ during *F. oxysporum* infection

**Figure 5:**
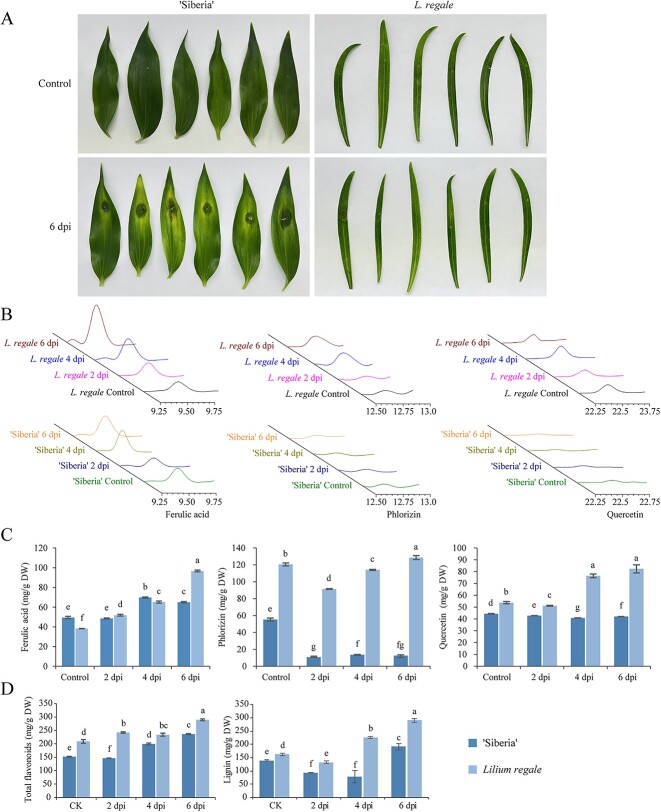
Accumulation of lignin, total flavonoids, ferulic acid, phlorizin, and quercetin in *L. regale* scales was greater and more rapid than in ‘Siberia’ during *F. oxysporum* infection. **A**  *L. regale* had higher resistance to *F. oxysporum* than lily ‘Siberia’. **B** HPLC chromatograms of ferulic acid, phlorizin, and quercetin in *L. regale* and ‘Siberia’ after inoculation with *F. oxysporum*. **C** Accumulation of ferulic acid, phlorizin, and quercetin in *L. regale* was higher than in ‘Siberia’. **D** Lignin and total flavonoid contents in *L. regale* were higher than in ‘Siberia’. Different lowercase letters indicated statistically significant differences between two samples (*P* < 0.05).

Multi-omics analysis revealed that the accumulation of phenylpropanes is crucial for the defense response of *L. regale* against *F. oxysporum*. To compare the accumulation of certain phenylpropanes during *F. oxysporum* infection, *L. regale* (resistant) and Oriental hybrid lily ‘Siberia’ (susceptible) were examined. At 6 dpi, ‘Siberia’ leaves exhibited severe chlorosis (yellowing), and the inoculation sites showed clear decay, consistent with the susceptibility of ‘Siberia’ to *F. oxysporum* (Fig. 5A). Subsequently, the lignin, total flavonoid, ferulic acid, phlorizin, and quercetin contents were determined in the scales of both lilies following *F. oxysporum* infection. HPLC chromatograms of ferulic acid, phlorizin, and quercetin (Fig. 5B) revealed significant differences in the accumulation of these phenylpropanes between *L. regale* and ‘Siberia’ scales. After inoculation with *F. oxysporum*, ferulic acid showed higher accumulation in *L. regale* compared with ‘Siberia’ (Fig. 5C). At 6 dpi, the ferulic acid content increased 2.53-fold in *L. regale*, while it only increased 1.31-fold in ‘Siberia’. Additionally, phlorizin content increased in *L. regale*, peaking at 6 dpi (128.53 mg/g dry weight), in contrast to the decrease observed in ‘Siberia’. Similarly, quercetin showed a significant increase (1.53-fold) in *L. regale* at 6 dpi, but decreased in ‘Siberia’. Regarding flavonoids, at 2 dpi the content was higher in *L. regale* (242.18 mg/g) compared with ‘Siberia’ (146.35 mg/g) (Fig. 5D), indicating the rapid accumulation of flavonoids in *L. regale* in response to *F. oxysporum* infection. At 6 dpi, lignin content increased 1.78-fold in *L. regale* (290.01 mg/g) (Fig. 5D), exceeding the lignin content in ‘Siberia’ (191.48 mg/g). Consequently, compared with ‘Siberia’, *F. oxysporum* infection led to a higher accumulation of phenylpropanes in *L. regale* scales.

### Phenylpropanes inhibited *F. oxysporum* growth and pathogenicity-related gene expression

**Figure 6:**
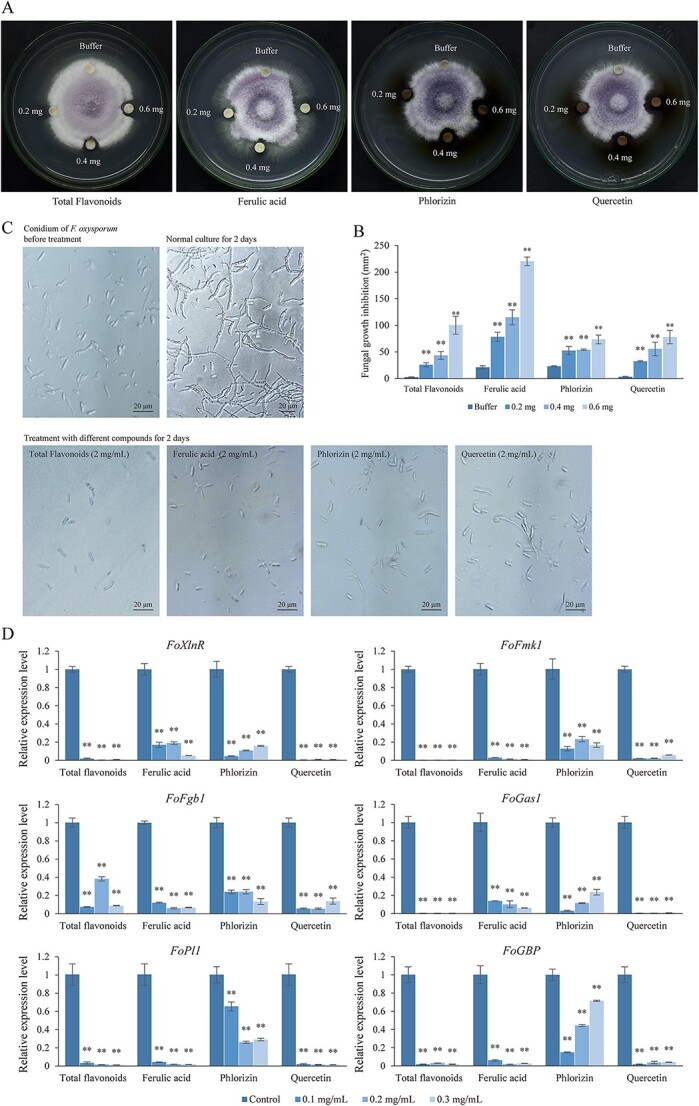
Total flavonoids, ferulic acid, phlorizin, and quercetin inhibited *F. oxysporum* growth*.*  **A** Total flavonoids, ferulic acid, phlorizin and quercetin inhibited the growth of *F. oxysporum*. **B** The growth inhibition area of *F. oxysporum *after treatment with total flavonoids of *L. regale*, ferulic acid, phlorizin, and quercetin, respectively. **C** Total flavonoids, ferulic acid, phlorizin, and quercetin inhibited conidial germination of *F. oxysporum*. **D** Expression levels of *F. oxysporum* genes required for pathogenicity were suppressed by total flavonoids, ferulic acid, phlorizin, and quercetin treatment. The *t*-test was used to reveal statistical differences between treatments and control (**P < 0.01).

The antifungal effects of the ferulic acid, phlorizin, quercetin, and total flavonoids extracted from *L. regale* were evaluated. The antifungal assay demonstrated that these phenylpropanes had significant inhibitory effects on *F. oxysporum* mycelial growth, with the inhibitory effects increasing as the concentrations of the compounds increased (Fig. 6A). Notably, 0.6 mg ferulic acid exhibited the most potent inhibition, resulting in a growth inhibition area of 220.11 mm^2^ (Fig. 6B). Even at smaller content (0.2 mg), flavonoids, ferulic acid, phlorizin, and quercetin inhibited *F. oxysporum* mycelial growth, with growth inhibition areas of 25.89, 78.49, 52.62, and 32.57 mm^2^, respectively. Furthermore, the addition of flavonoids, ferulic acid, phlorizin, and quercetin (2 mg/mL) to the liquid culture medium adversely affected the germination of *F. oxysporum* conidia (Fig. 6C).

The expression of *F. oxysporum* pathogenicity-related genes was analyzed after supplementing the culture medium with total flavonoids, ferulic acid, phlorizin, and quercetin (0.1, 0.2, and 0.3 mg/mL for all treatments). The treatments with these compounds led to the suppression of pathogenicity-related gene expression in *F. oxysporum* (Fig. 6D). For instance, treatment with 0.2 mg/L total flavonoids significantly downregulated the expression of *xylanase transcriptional activator* (*FoXlnR*), which influences the growth of saprophytic and pathogenic *F. oxysporum*. The expression levels of two *F. oxysporum* virulence genes, *mitogen-activated protein kinas*e (*FoFmk1*) and *guanine nucleotide-binding protein* (*FoFgb1*), were only 0.2 and 6.7% of the corresponding control levels after treatment with 0.3 mg/L total flavonoids and ferulic acid, respectively. The expression of two plant cell wall degradation-related genes, *beta-1,3-glucanosyltransferase* (*FoGas1*) and *pectate lyase* (*FoPl1*), was downregulated by treatment with 0.2 and 0.3 mg/L total flavonoids, with expression levels reaching only 0.3 and 1.2% of the corresponding control levels, respectively. Moreover, 0.1 mg/L quercetin treatment significantly inhibited the expression of *F. oxysporum GTPase-binding protein* (*FoGBP*).

### Transcriptional network regulating phenylpropanoid biosynthesis during the *L. regale* defense response to *F. oxysporum*

**Figure 7:**
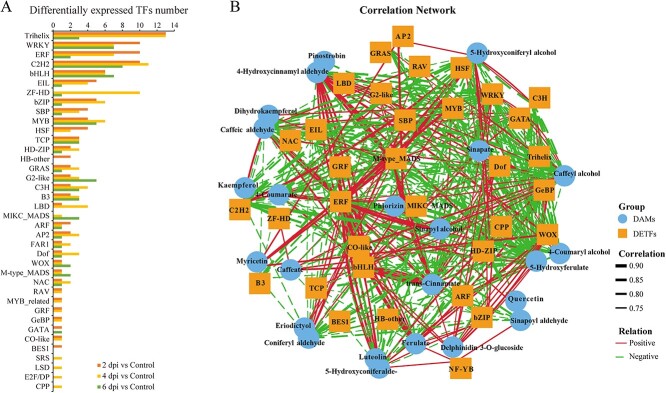
Transcription regulatory network analysis of phenylpropanes responding to *F. oxysporum*. **A** Identification of differentially expressed transcription factors at 2, 4, and 6 dpi. **B** Transcription regulator network of phenylpropanes according to multi-omic integrative analysis.

TFs play a crucial role in regulating defense responses in plants. To investigate the transcriptional regulation of phenylpropanoid biosynthesis, we analyzed the correlation between phenylpropane contents determined by multi-omics analysis and the expression patterns of differentially expressed transcription factors (DETFs) identified from the transcriptomics data. A total of 1526 unigenes were identified as TFs, with 104, 114, and 67 DETFs found to be differentially expressed at 2, 4, and 6 dpi, respectively. These DETFs belonged to 38 TF families, including Trihelix, MYB, bZIP (basic region leucine zipper), and GRF (growth-regulating factor) families (Fig. 7A). The majority of DETFs belonged to the Trihelix, WRKY, ERF, and C2H2 families.

Additionally, 34 TFs were found to regulate 24 phenylpropane synthesis pathways (Fig. 7B). Among them, ERF, bZIP, and WRKY family members were found to regulate the synthesis of 16, 15, and 13 phenylpropanes, respectively, with a Pearson correlation coefficient (ρ) of >0.7 or <−0.7. TFs with a ρ value >0.8 or <−0.8 are listed in Table 1. The ERF family was found to positively regulate the synthesis of phenylpropanes such as phlorizin and ferulic acid, based on the high ρ value (0.85) for the correlation between ferulic acid/5-hydroxyconiferaldehyde and ERF. Additionally, one glabrous-enhancer-binding protein (GeBP) was predicted to modulate sinapic acid biosynthesis. On the other hand, the WRKY family was predicted to negatively regulate the biosynthesis of phenylpropanes such as 4-coumarate, 4-hydroxycinnamyl aldehyde, 5-hydroxyferulic acid, caffeic aldehyde, caffeyl alcohol, and *trans*-cinnamate. Furthermore, the C2H2 family was predicted to negatively regulate the biosynthesis of 4-coumaryl alcohol/5-hydroxyconiferyl alcohol.

**Table 1:** Correlation analysis between differentially expressed transcription factors and differentially accumulated phenylpropanes in *L. regale*

**Differentially expressed transcription factors**	**Differentially accumulated phenylpropanes**	**Pearson correlation coefficient** **>0.8 or <−0.8**	***P*-value**	**Relation**	**Differentially expressed transcription factors**	**Differentially accumulated phenylpropanes**	**Pearson correlation coefficient** **<−0.8**	***P*-value**	**Relation**
C2H2	5-Hydroxyconiferyl alcohol	0.818	0.0011	Positive	EIL	Caffeyl alcohol	−0.882	0.0001	Negative
gERF	*trans*-Cinnamate	0.832	0.0007		CPP	Coniferyl aldehyde	−0.850	0.0004	
ERF	Ferulate	0.859	0.0003		GeBP	Ferulate	−0.810	0.0013	
ERF	Sinapyl alcohol	0.863	0.0002		GeBP	5-Hydroxyconiferaldehyde	−0.810	0.0013	
ERF	Caffeic aldehyde	0.842	0.0005		GRAS	Sinapoyl aldehyde	−0.866	0.0002	
ERF	Caffeic aldehyde	0.810	0.0013		HB-other	5-Hydroxyconiferyl alcohol	−0.806	0.0015	
ERF	5-Hydroxyconiferyl alcohol	0.835	0.0007		HSF	5-Hydroxyferulate	−0.837	0.0006	
ERF	Phlorizin	0.835	0.0067		M-type_MADS	5-Hydroxyferulate	−0.827	0.0008	
ERF	5-Hydroxyconiferyl alcohol	0.822	0.0010		MYB	Caffeyl alcohol	−0.809	0.0014	
ERF	5-Hydroxyconiferaldehyde	0.859	0.0003		MYB	5-Hydroxyferulate	−0.861	0.0003	
ERF	4-Hydroxycinnamyl aldehyde	0.832	0.0007		TCP	5-Hydroxyferulate	−0.813	0.0012	
ERF	4-Coumarate	0.842	0.0005		TCP	5-Hydroxyconiferyl alcohol	−0.822	0.0010	
ERF	4-Coumarate	0.810	0.0013		Trihelix	Coniferyl aldehyde	−0.831	0.0007	
GeBP	Sinapate	0.888	0.0001		Trihelix	Caffeyl alcohol	−0.850	0.0004	
M-type_MADS	*trans*-Cinnamate	0.886	0.0001		WRKY	trans-Cinnamate	−0.815	0.0012	
M-type_MADS	5-Hydroxyconiferyl alcohol	0.813	0.0012		WRKY	Caffeyl alcohol	−0.820	0.0010	
M-type_MADS	4-Hydroxycinnamyl aldehyde	0.886	0.0001		WRKY	Caffeic aldehyde	−0.805	0.0015	
M-type_MADS	4-Hydroxycinnamyl aldehyde	0.817	0.0011		WRKY	Caffeic aldehyde	−0.810	0.0013	
MYB	Myricetin	0.819	0.0011		WRKY	5-Hydroxyferulate	−0.881	0.0001	
B3	5-Hydroxyconiferyl alcohol	−0.829	0.0008	Negative	WRKY	4-Hydroxycinnamyl aldehyde	−0.815	0.0012	
C2H2	5-Hydroxyferulate	−0.812	0.0013		WRKY	4-Coumarate	−0.805	0.0015	
C2H2	5-Hydroxyconiferyl alcohol	−0.913	0.0000		WRKY	4-Coumarate	−0.810	0.0013	
C2H2	4-Coumaryl alcohol	−0.913	0.0000		C3H	5-Hydroxyferulate	−0.813	0.0012	
EIL	Caffeyl alcohol	−0.828	0.0008		C3H	5-Hydroxyconiferyl alcohol	−0.800	0.0017	

### Positive regulatory effects of LrERF4 on *L. regale* resistance to *F. oxysporum*

**Figure 8:**
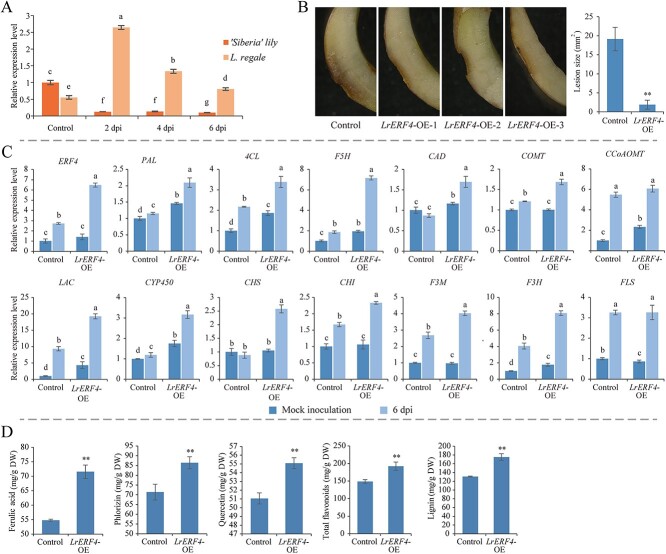
LrERF4 enhanced the resistance of ‘Siberia’ to *F. oxysporum* by upregulating the biosynthesis of lignin, total flavonoids, ferulic acid, phlorizin, and quercetin. **A**  *ERF4* expression patterns were significantly different in *L. regale* and ‘Siberia’ during *F. oxysporum* infection. **B** Susceptibility of lily ‘Siberia’ scales to *F. oxysporum* reduced after *LrERF4* overexpression, which was evidenced by the decreased rot area compared with the control. **C** Expression of genes related to biosynthesis of lignin, total flavonoids, ferulic acid, phlorizin, and quercetin was upregulated in ‘Siberia’ scales undergoing *LrERF4* overexpression. **D** Contents of lignin, total flavonoids, ferulic acid, phlorizin, and quercetin in ‘Siberia’ scales increased after *LrERF4* overexpression. The *t*-test was used to reveal statistical differences between different samples (**P < 0.01). Different lowercase letters indicated statistically significant differences between two samples (*P* < 0.05).

The above-mentioned analysis of the transcriptional regulatory network suggests that ERF TFs likely have a positive regulatory role in the biosynthesis of phenylpropanes, such as flavonoids and hydroxycinnamic acid. Therefore, we cloned and characterized an *ERF* gene from *L. regale* (*LrERF4*) to investigate its function. We also examined the expression profiles of *ERF4* in *F. oxysporum*-infected *L. regale* and lily ‘Siberia’ plants. Compared with the control, the expression of *LrERF4* increased 2.5-fold at 2 dpi, while the expression level of *ERF4* decreased in *F. oxysporum*-infected ‘Siberia’ (Fig. 8A). This suggests that a rapid increase in *LrERF4* expression may contribute to the resistance of *L. regale* to *F. oxysporum*.

Furthermore, we transiently overexpressed *LrERF4* in lily ‘Siberia’ scales and inoculated them with *F. oxysporum*. The average lesion area on the *LrERF4*-overexpressing (OE) scales (1.88 mm^2^) was only 9% of that on the control scales containing the empty vector (Fig. 8B). This indicated that the transient overexpression of *LrERF4* reduced the susceptibility of ‘Siberia’ to *F. oxysporum*. Quantitative real-time PCR (qPCR) analysis confirmed the expression of *LrERF4* in the ‘Siberia’ scales (Fig. 8C). The expression of the *PAL* gene increased by 1.45-fold in the *LrERF4*-OE scales. Additionally, the lignin synthesis-related genes *4-coumarate-CoA ligase* (*4CL*), *cytochrome P450* (*CYP450*), *LAC*, and c*affeoyl-CoA O-methyltransferase* (*CCoAOMT*) were upregulated in the *LrERF4*-OE scales (1.85-, 1.74-, 4.29-, and 2.31-fold, respectively). The expression of the flavonoid biosynthesis-related genes *naringenin 3-dioxygenase* (*F3H*) and *CHS* was upregulated by 1.76- and 1.05-fold in the *LrERF4*-OE scales, respectively. Furthermore, the expression of *F5H*, which contributes to hydroxycinnamic acid biosynthesis, increased by 1.93-fold in the *LrERF4*-OE scales. The lignin, total flavonoid, ferulic acid, phlorizin, and quercetin contents also increased in the *LrERF4*-OE scales (Fig. 8D). Specifically, the lignin and total flavonoid contents increased by 1.33- and 1.29-fold, respectively. The accumulation levels of ferulic acid, phlorizin, and quercetin in ‘Siberia’ scales overexpressing *LrERF4* increased by 1.30-, 1.21-, and 1.07-fold, respectively. Therefore, it appears that *LrERF4* positively regulates the biosynthesis of phenylpropanes, including lignin, flavonoids, ferulic acid, phlorizin, and quercetin.

**Figure 9:**
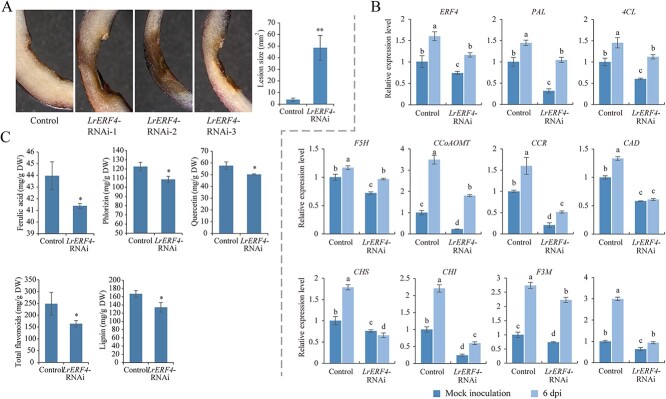
Resistance of *L. regale* was downregulated after *LrERF4* RNAi, accompanied by decreased lignin, total flavonoid, ferulic acid, phlorizin, and quercetin contents. **A** Resistance of *L. regale* scales to *F. oxysporum* was damaged after expression of *LrERF4* RNAi vector, which was supported by the increased rot area of *F. oxysporum* infection. **B** Expression of genes related to lignin, total flavonoids, ferulic acid, phlorizin, and quercetin biosynthesis was suppressed in *L. regale* scales by *LrERF4* RNAi. **C** Accumulation of lignin, total flavonoids, ferulic acid, phlorizin, and quercetin in *L. regale* scales decreased after *LrERF4* RNAi. The *t*-test was used to reveal statistical differences between samples (*P < 0.05, **P < 0.01). Different lowercase letters indicated statistically significant differences between two samples (*P* < 0.05).

To suppress the expression of *LrERF4* in *L. regale*, the RNAi vector pHellsgate2-*LrERF4* was introduced into *L. regale* scales using *Agrobacterium tumefaciens*. After 72 h, the *L. regale* scales were infected with *F. oxysporum*, and the disease symptoms were observed at 6 dpi. Compared with the control *L. regale* scales expressing the empty RNAi vector, the resistance of *L. regale* scales expressing pHellsgate2-*LrERF4* was reduced (Fig. 9A), with an average lesion area 12.65-fold larger than that of the control scales. QPCR analysis confirmed that the expression of *LrERF4* decreased in the scales transformed with pHellsgate2-*LrERF4* (Fig. 9B). This decreased expression resulted in the downregulation of phenylpropanoid biosynthesis-related genes. Notably, the relative expression levels of genes involved in lignin/lignan biosynthesis, such as *PAL*, *4CL*, *F5H*, *CCoAOMT*, *CCR*, and *coniferyl-alcohol dehydrogenase* (*CAD*), decreased by 72, 77, 82, 51, 32, and 45%, respectively, compared with the corresponding control levels. The relative expression of flavonoid biosynthesis-related genes, including *CHI*, *F3M*, and *F3H*, decreased to 27, 81, and 31% of the corresponding control levels, respectively, following the RNAi-mediated silencing of *LrERF4*. Moreover, the lignin, total flavonoid, ferulic acid, phlorizin, and quercetin contents decreased to 80, 66, 94, 88, and 87% of the corresponding control levels, respectively, after the silencing of *LrERF4* by RNAi (Fig. 9C).

## Discussion

In plants, broad-spectrum and long-lasting resistance to stress, known as QTR, is mediated by genes related to defense responses, metabolite production, and phytohormone production [26, 27]. In *Z. mays*, *ZmCCoAOMT2* and *ZmCAD*, which are involved in lignin biosynthesis, were confirmed to encode components of the QTR mechanism protecting against leaf diseases [28, 29]. As a highly resistant wild lily, *L. regale* is valuable for breeding disease-resistant lily varieties [30]. Although previous research has identified several WRKY TFs that positively regulate *L. regale* resistance to fusarium wilt, the QTR mechanism has not been thoroughly investigated. This study was conducted to characterize the QTR mechanism that protects *L. regale* from *F. oxysporum*, thus providing insights into the molecular basis of its resistance.

Multi-omics techniques are useful for comprehensively exploring plant defense mechanisms activated by pathogens. Integrated transcriptome and proteome analysis has confirmed the importance of phenylpropanoid biosynthesis and linoleic acid metabolism in the resistance of *Z. mays* to *F. verticillioides* [31]. Additionally, protein-lysine 6-oxidase 2 was found to positively regulate maize resistance to *F. verticillioides* through the salicylic acid-mediated pathway [31]. In this study, multi-omics analysis revealed that pathways involved in phenylpropanoid, flavonoid, and isoquinoline alkaloid biosynthesis were rapidly differentially expressed during early *F. oxysporum* infection (2 dpi) (Figs 1B, 2D and 3B). The flavone and flavonol biosynthesis pathways were also differentially expressed during the interaction between *L. regale* and *F. oxysporum* (4 dpi), as were the phenylpropanoid, flavonoid, and isoquinoline alkaloid biosynthesis pathways (Figs 1C, 2E, and 3C). In the late *F. oxysporum* infection stage (6 dpi), the phenylpropanoid and flavonoid biosynthesis pathways were still differentially expressed according to the multi-omics data (Figs 1D, 2F, and 3D). These pathways, along with glutathione metabolism, oxidative phosphorylation, plant hormone signal transduction, and MAPK signaling, were significantly altered by *F. oxysporum* infection (Figs 1B–D and 2D–F). These results suggested that phenylpropane metabolism, hormone signaling, and MAPK signaling contribute to the resistance of *L. regale* to fusarium wilt.

In addition to phenylpropanes, other secondary metabolites, such as monoterpenoids and isoquinoline alkaloids, play important roles in plant defense responses [32, 33]. The biosynthesis of monoterpenoids was found to increase during *Ophiostoma bicolor* infection in *Picea koraiensis* [34]. A monoterpenoid called citral enhanced the resistance of *N. benthamiana* to tobacco mosaic virus [35]. Metabolomics analysis has shown that certain metabolites, like l-dopa and tyramine, accumulate more in resistant *T. aestivum* compared with a susceptible strain during *Blumeria graminis* infection [36]. Thalicfoetine isolated from *Thalictrum foetidum* was found to inhibit *Bacillus subtilis* growth [37]. The multi-omics analysis conducted in the current study revealed that, in addition to phenylpropane production, the biosynthesis of monoterpenoids (geranylgeranyl diphosphate) and isoquinoline alkaloids (dopamine) might play an important role during the interaction between *L. regale* and *F. oxysporum* (Figs 2–4 and Supplementary Data Figs S1 and S2). These results reflected that the regulation by secondary metabolism of resistance of *L. regale* to fusarium wilt is complex, and indicate that the contributions of these regulatory networks to the defense response against *F. oxysporum* need future studies.

Pathogenic fungal infections in plants lead to the accumulation of resistance-related metabolites such as phenylpropanes. Phenylpropanes derived from phenylalanine are mainly divided into the precursors of lignin and lignans, flavonoids and hydroxycinnamic acid, which play essential roles associated with plant development and disease resistance [38]. The metabolome analysis in this study revealed an increase in phenylpropanoid content in *F. oxysporum*-resistant *L. regale* at different time points (Fig. 4). Further analysis confirmed the accumulation of phenylpropanes, such as lignin, flavonoids, ferulic acid, phlorizin, and quercetin, during *F. oxysporum* infection of *L. regale*, with ‘Siberia’ serving as a control (Fig. 5). During *F. oxysporum* infection, the abundance of these phenylpropanes changed considerably less in ‘Siberia’ than in *L. regale*. Total flavonoids, ferulic acid, phlorizin, and quercetin showed inhibitory effects on *F. oxysporum* growth and the expression of pathogenicity-related genes (Fig. 6). Therefore, it appears that the activation of phenylpropanoid metabolism is crucial for the QTR mechanism that protects *L. regale* from fusarium wilt.

The phenylpropanoid metabolism-based defense response to pathogens has been partially characterized in plants. In *Oryza sativa*, MYB30 activates lignin biosynthesis genes, resulting in increased lignin accumulation and resistance to *Magnaporthe oryzae* [39]. The application of certain phenylpropanes, such as kaempferide and apigenin, enhances the resistance of wheat to *F. graminearum* [40]. Chlorogenic acid, a hydroxycinnamic acid derivative, restricts *Fusarium fujikuroi* growth by inducing a burst of reactive oxygen species [41]. Flavonoids can disrupt the plasma membrane of pathogens, disrupt mitochondrial functions, and inhibit cell wall formation, all of which have detrimental effects on pathogen growth [42]. In this study, the total flavonoids, ferulic acid, phlorizin, and quercetin extracted from *L. regale* exhibited inhibitory effects on *F. oxysporum* growth and the expression of pathogenicity-related genes (Fig. 6). These results indicated that the phenylpropanes that accumulate in *L. regale* contribute to the physical and chemical defense barriers during *F. oxysporum* infection.

TFs play a crucial role in the transcriptional reprogramming of plants during pathogen infections, influencing the accumulation of resistance-related metabolites. *MdMYB1r1* positively regulates the expression of *MdBGLU40*, promoting the synthesis of coumarin, which inhibits *Cytospora mali* infection [43]. In *Gossypium hirsutum* infected with *F. oxysporum*, VIIIc WRKY TFs activate *GhMKK1* expression, leading to the biosynthesis of flavonoids through an MAPK cascade involving GhNTF6 and GhMYC2 [44]. *Chrysanthemum morifolium* WRKY6-1 negatively regulated chrysanthemum resistance to *F. oxysporum* by reducing salicylic acid biosynthesis, inducing reactive oxygen species production and inhibiting *CmWRKY15-like* expression [45]. In *Vitis davidii*, MYB1 upregulates *VdSTS2* expression, increasing resveratrol content and resistance to *Erysiphe necator* [46]. In this study, our analysis of the transcriptional regulatory network suggested that ERF, bZIP, and WRKY TFs might regulate phenylpropanoid biosynthesis (Fig. 7). Through reverse genetics, we found that *LrERF4* regulates the accumulation of phenylpropanes during *F. oxysporum* infection (Figs 8 and 9). Transient overexpression of *LrERF4* protected ‘Siberia’ from *F. oxysporum* by increasing the contents of lignin, total flavonoids, ferulic acid, phlorizin, and quercetin. Conversely, RNAi-mediated suppression of *LrERF4* expression in *L. regale* prevented the accumulation of these phenylpropanes and decreased resistance to *F. oxysporum*. Therefore, LrERF4 positively regulates the resistance of *L. regale* to fusarium wilt by modulating the biosynthesis of defense-related phenylpropanes.

## Conclusion

This study analyzed the transcriptome, proteome, and metabolome profiles of *L. regale* in response to *F. oxysporum*. The integrated multi-omics analysis revealed the importance of phenylpropanoid metabolism in protecting *L. regale* from *F. oxysporum*. Moreover, some flavonoids and hydroxycinnamic acids were more abundant in resistant* L. regale* compared with susceptible ‘Siberia’. These phenylpropanes showed inhibitory effects on* F. oxysporum* growth, conidial germination, and pathogenicity-related gene expression, suggesting their contribution to the chemical and physical defenses of *L. regale*. Furthermore, LrERF4 was identified as a key TF regulating the biosynthesis of defense-related phenylpropanes in *L. regale*. The findings highlight the importance of phenylpropanoid metabolism in the high-level resistance of *L. regale* to Fusarium wilt.

## Materials and methods

### Lily materials and *F. oxysporum* inoculation

*Lilium regale* and Oriental hybrid lily ‘Siberia’ plants were cultivated in a greenhouse. ‘Siberia’ plants showing symptoms of fusarium wilt were used to isolate *F. oxysporum*, which was then preserved in our laboratory [47]. Prior to use, *F. oxysporum* was cultured on potato dextrose agar (PDA).

*Fusarium oxysporum* conidial suspension (5 × 10^6^ conidia/mL) was used to infect healthy *L. regale* plants by dipping the roots. Plants subjected to mock inoculation with sterile water were used as controls. At 6 h post-inoculation, the plants were transferred to sterile soil. The roots were collected at 2, 4, and 6 dpi for omics analysis, with three biological replicates.

### RNA isolation, sequencing, and transcriptome analysis

Total RNA was extracted from *L. regale* roots (sample collection is described in section ‘Lily materials and *F. oxysporum* inoculation’) to isolate mRNA. The mRNA was fragmented, and a cDNA library was synthesized and sequenced using the Illumina NovaSeq™ 6000 system (LC Sciences, USA).

High-quality clean sequencing reads were selected using Cutadapt (v.1.9.1), and *de novo* assembly of the high-quality reads was performed using Trinity (v.2.4.0) [48]. The longest sequence in each cluster was selected as the unigene. The unigenes were aligned with sequences in the KEGG and eggNOG databases using DIAMOND [49]. Salmon (v.0.8.2) [50] was used to analyze unigene expression levels. Significant DEGs (fold change >1.5 or <0.5 relative to control levels and *P* < 0.05) were obtained using the R package edgeR [51]. KOBAS was used for KEGG enrichment analysis of DEGs.

### Protein extraction, annotation, and proteome analysis

The concentration of protein extracted from *L. regale* roots (sample collection is described in section ‘Lily materials and *F. oxysporum *inoculation’) was determined using a BCA kit (Beyotime, China). The protein samples were then treated with dithiothreitol and iodoacetamide. After precipitation and washing with acetone, peptides were obtained by trypsin digestion. The peptides were labeled using TMTpro™ 16plex Label Reagent (Thermo, USA) and pre-separated using the LC-20AD system (Shimadzu, Japan). The labeled peptides were analyzed as described in Liu *et al*. [52].

Proteome Discoverer software was used for protein identification, which was subsequently annotated in the KEGG database. Significant DEPs (fold change >1.5 or <0.5 relative to control levels and *P* < 0.05) were mapped to KEGG pathways using KEGG Mapper. Enriched pathways were identified using a two-tailed Fisher’s exact test.

### Metabolite extraction, determination, and metabolome analysis

Metabolites were extracted from *L. regale* roots (sample collection is described in section ‘Lily materials and *F. oxysporum *inoculation’) for UPLC–MS/MS analysis. Separation and detection of metabolites were performed according to the methods described in Liu *et al*. [52]. The acquired MS data were processed to obtain retention time and exact mass for identification of ions. Metabolites were annotated by matching the *m*/z values of samples with those in the KEGG database.

Metabolome data were statistically analyzed as described in Liu *et al*. [53]. The DAMs (fold change >1.5 or <0.5 relative to control levels) were classified based on phytochemical compounds in the KEGG database. KOBAS was used to identify enriched pathways.

### Inoculation and collection of *L. regale* and ‘Siberia’ scales for HPLC analysis of metabolite contents

Differences in phenylpropane accumulation between ‘Siberia’ and *L. regale* were examined. *Fusarium oxysporum* conidial suspension (1 × 10^6^ conidia/mL) was used to infect leaves of ‘Siberia’ and *L. regale*, and fungal infection damage was recorded at 6 dpi. Additionally, healthy *L. regale* and ‘Siberia’ plants were infected with *F. oxysporum* conidial suspension (5 × 10^6^ conidia/mL), and scales were harvested at 2, 4, and 6 dpi for analysis of lignin, total flavonoid, ferulic acid, phlorizin, and quercetin contents as described in section ‘Analyses of lignin, total flavonoid, ferulic acid, phlorizin, and quercetin contents’.

### Antifungal effects of total flavonoids, luteolin, and quercetin extracted from *L. regale*

To obtain flavonoids, dried *L. regale* scale samples (1 g) were added to a 25-mL NaOH solution (pH 10) for a 45-min ultrasonic extraction at 60°C. After cooling the supernatant the extract was harvested through centrifugation, and the pH of the solution was adjusted to 3.5 before incubation for 12–16 h. After centrifugation the supernatant was removed, and the sediment was washed with ether and distilled water. Total flavonoids were dissolved in methanol after the sediment had been dried to a constant weight.

After pre-culturing *F. oxysporum* on PDA to a fungal colony diameter of 2 cm, four sterile paper discs were evenly placed on the periphery of *F. oxysporum* colonies, and the angle between two neighbouring discs was 90°. The inhibitory effects of the total flavonoid, ferulic acid, phlorizin, and quercetin solutions on *F. oxysporum* mycelial growth were examined in separate plates. More specifically, the following reagents were added to the discs: 40 μL flavonoid solution (5, 10, and 15 mg/mL), 40 μL ferulic acid solution (5, 10, and 15 mg/mL), 40 μL phlorizin solution (5, 10, and 15 mg/mL), or 40 μL quercetin solution (5, 10, and 15 mg/mL). We added 40 μL methanol to the control disc in each plate. Fungal growth was recorded after incubation at 28°C for 4 days using a digital camera (Nikon, Japan), and then Photoshop 7.0 was used to analyze the effects of various solutions on fungal growth. The antifungal assay was repeated three times.

After pre-culturing *F. oxysporum* on PDA for 5 days, an *F. oxysporum* conidial suspension was prepared using potato dextrose broth. The conidial concentration was adjusted to 1 × 10^5^ conidia/mL, after which 200 μL of conidial suspension was mixed with 40 μL total flavonoid, ferulic acid, phlorizin, or quercetin solution (initial concentration 10 mg/mL). A mixture comprising the conidial suspension and 40 μL methanol served as the control. The germination of *F. oxysporum* conidia after incubation at 28°C for 24 h was observed using an inverted microscope (Leica, Germany). The conidial germination assay was repeated three times.

In addition, *F. oxysporum* mycelia were collected from PDA plates after the 4-day treatment with the flavonoid, ferulic acid, phlorizin, quercetin, and methanol (control) solutions to determine the expression of pathogenicity-related genes in *F. oxysporum* by qPCR. The 2^−ΔΔCt^ method was employed to obtain the values of gene expression with an internal reference gene (*F. oxysporum β-actin*, GenBank No. XM_031177127). The qPCR primers are listed in Supplementary Data Table S2, and this experiment included three biological replicates and three technical replicates.

### Transcriptional regulatory network analysis

Unigene sequences were aligned with the TF sequences from *O. sativa* and *A. thaliana* in the PlantDB database to identify TFs. The DETFs (fold change >1.5 or <0.5 relative to control levels and *P* < 0.05) were classified using the PlantDB database. The OmicStudio tools were used to construct transcriptional regulatory networks for the DETFs and DAMs. Significant regulatory relationships between DETFs and DAMs were determined based on a ρ value >0.70 or <−0.70 and *P* < 0.05.

### Gene function analysis of *LrERF4*

To analyze the expression patterns of *ERF4* in *L. regale* and ‘Siberia’, their roots were collected after *F. oxysporum* (5 × 10^6^ conidia/mL) inoculation for 2, 4, and 6 days. Total RNA was extracted using the TRIGene kit (Genstar, China), and cDNA was synthesized for qPCR analysis with *ERF4*-specific primers (Supplementary Data Tables S3 and S4). The expression values of *ERF4* were calculated using the 2^−ΔΔCt^ method, with glyceraldehyde-3-phosphate dehydrogenase genes of *L. regale* and ‘Siberia’ as internal reference genes.

The *LrERF4* open reading frame (supplementary material ‘The cDNA sequence of *L. regale* and ‘Siberia’ lily ethylene-responsive transcription factor 4’) was cloned and used to construct pCAMBIA2300S-*LrERF4* overexpression vector and pHellsgate2-*LrERF4* RNA interference vector according to the methods in Su *et al.* [54]. The recombinant vectors were insterted into *A. tumefaciens* for genetic transformation of ‘Siberia’ and *L. regale* scales.

The scales and leaves of *L. regale* and ‘Siberia’ showed clear disease symptoms at 6 dpi. Meanwhile, the efficiency of transient RNAi/overexpression technology was high at 48–72 h after the *A. tumefaciens*-mediated transformation. Therefore, the scale samples were collected after pCAMBIA2300S-*LrERF4* and pHellsgate2-*LrERF4* were transiently expressed for 72 h to analyze the metabolite contents and gene expression levels as described in sections ‘Analyses of lignin, total flavonoid, ferulic acid, phlorizin, and quercetin contents’ and ‘Antifungal effects of total flavonoids, luteolin, and quercetin extracted from *L. regale*’, respectively. The primers for the qPCR analysis (Supplementary Data Tables S3 and S4) were designed according to the *L. regale* and ‘Siberia’ transcriptome data. Scales transformed with empty vectors were used as controls. In addition, the *L. regale* and ‘Siberia’ scales undergone transient RNAi/overexpression for 72 h were further inoculated with *F. oxysporum*, and at 6 dpi, all samples were harvested for evaluation of resistance and gene expression. The disease symptoms on the transgenic *L. regale* and ‘Siberia’ scales were recorded using a stereomicroscope (Olympus, Japan). These experiments included three biological replicates.

### Analyses of lignin, total flavonoid, ferulic acid, phlorizin, and quercetin contents

Lily scales (sample collection is described in sections ‘Inoculation and collection of *L. regale* and ‘Siberia’ scales for HPLC analysis of metabolite contents’ and ‘Gene function analysis of *LrERF4*’) were dried to a constant weight. The content of lignin was determined as in the protocol described by Wadenbäck *et al.* [55]. Flavonoids were extracted from 0.01 g dried scale samples using ethanol solution and then mixed with Al(NO_3_)_3_ solution under alkaline conditions. The content of total flavonoid was determined using a multimicroplate reader (SpectraMax M2, USA) with absorbance at 470 nm. Rutin (Yuanye, China) was used to construct a standard curve for determination of total flavonoids. These experiments included three biological replicates.

To determine the ferulic acid, phlorizin, and quercetin contents, 0.5 g dried scale samples were added to a 15-mL 60% CH_3_OH solution for a 90-min ultrasonic extraction at 60°C. The extract was centrifuged and then concentrated using a rotary evaporator (N-1300, Eyela, Japan). The concentrated solution was used for HPLC analysis by an LC-40A system equipped with a Unitary C18 column (Shimadzu, Japan). There were three biological replicates included in this experiment.

### Statistical analysis

Statistical differences in *ERF4* expression level and contents of lignin, total flavonoids, ferulic acid, phlorizin, and quercetin in *L. regale* and ‘Siberia’ after *F. oxysporum* inoculation were calculated by a multiple *t*-test. Student’s *t*-test was used to analyze the antifungal effects of the total flavonoids, ferulic acid, phlorizin, and quercetin on *F. oxysporum* growth. Student’s *t*-test was also used to analyze the expression levels of pathogenicity-related genes after treatment with the different metabolites. The expression levels of genes related to *LrERF4* and phenylpropane biosynthesis as well as the lignin, total flavonoid, ferulic acid, phlorizin, and quercetin contents in *L. regale* after *LrERF4* RNAi and in ‘Siberia’ after *LrERF4* overexpression were also determined by Student’s *t*-test.

## Supplementary Material

Web_Material_uhae140
