# Supplementary material for: Integrated multi-omics investigation revealed the importance of phenylpropanoid metabolism in the defense response of Lilium regale Wilson to fusarium wilt
Source: Hortic Res. 2024 May 20;11(7):uhae140. doi: 10.1093/hr/uhae140 (PMC11233880; doi:10.1093/hr/uhae140)
Supplement: Web_Material_uhae140 [file web_material_uhae140.zip › Supplementary data.docx]

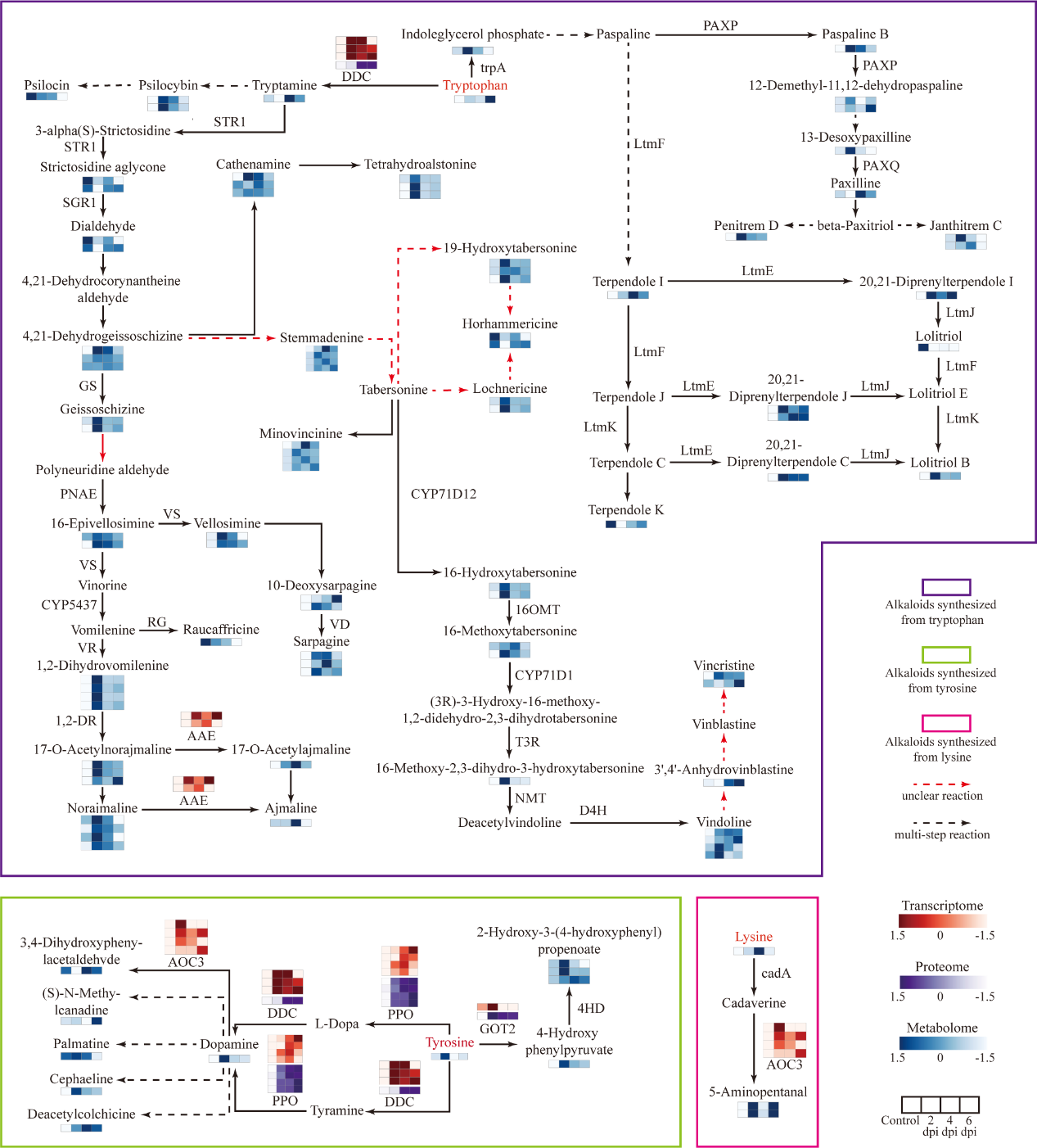


**Figure. S1** The multi-osmic integrative analysis of alkaloids biosynthesis in *L. regale* during *F. oxysporum* infection. The synthesis of alkaloids synthesized from tryptophan, alkaloids synthesized from tyrosine, alkaloids synthesized from lysine were framed by purple, green and pink lines, respectively. The black dashed line represents a multi-step reaction. The red dashed line represents an unknown reaction. PAXP/PAXQ, cytochrome P450 monooxygenase; trpA, tryptophan synthase alpha chain; DDC, aromatic-L-amino-acid/L-tryptophan decarboxylase; STR1, strictosidine synthase 1; SGR1, strictosidine beta-D-glucosidase 1; GS, geissoschizine synthase; PNAE, polyneuridine-aldehyde esterase; VS, vinorine synthase; VR, vomilenine reductase; 1,2-DR, 1,2-dihydrovomilenine reductase; AAE, acetylajmaline esterase; VD, vellosimine dehydrogenase; CYP71D12, tabersonine 16-hydroxylase; 16OMT, tabersonine 16-O-methyltransferase; CYP71D1, tabersonine 3-oxygenase; T3R, 3-hydroxy-1,2-didehydro-2,3-dihydrotabersonine reductase; NMT, 3-hydroxy-16-methoxy-2,3-dihydrotabersonine N-methyltransferase; D4H, deacetoxyvindoline 4-hydroxylase; LtmF, dimethylallyldiphosphate transferase; LtmE, dimethylallyldiphosphate transferase; LtmJ, cytochrome P450 monooxygenase; LtmK, cytochrome P450 monooxygenase; PAXQ, cytochrome P450 monooxygenase; AOC3, primary-amine oxidase; DDC, aromatic-L-amino-acid/L-tryptophan decarboxylase; GOT2, aspartate aminotransferase, mitochondria; PPO, polyphenol oxidase ; 4HD, 4-hydroxyphenylpyruvate decarboxylase; cadA, lysine decarboxylase.


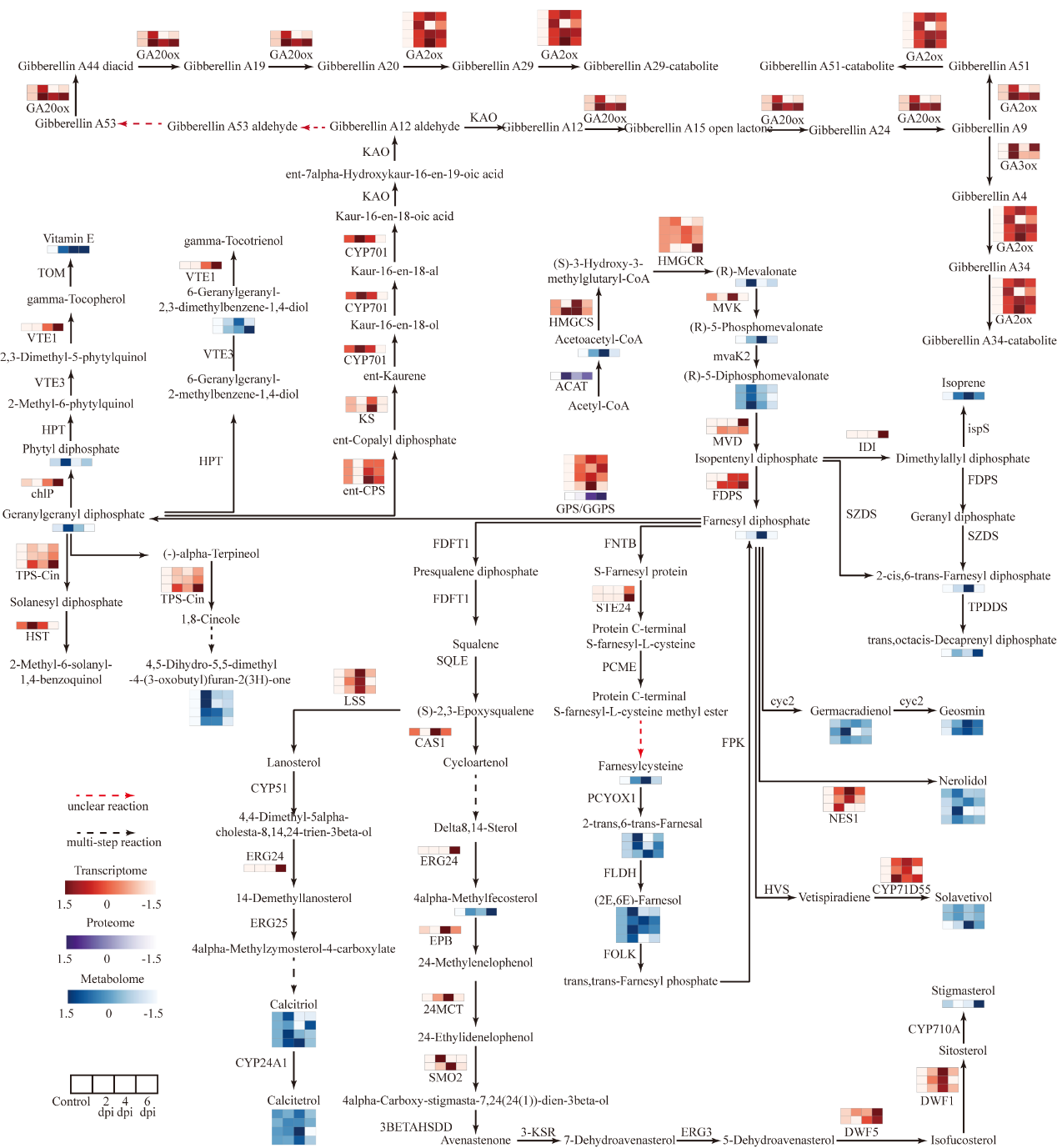


**Figure. S2** The multi-osmic integrative analysis of terpenoids biosynthesis in *L. regale* during *F. oxysporum* infection. The black dashed line represents a multi-step reaction. The red dashed line represents an unknown reaction. FDPS, farnesyl diphosphate synthase; MVD, diphosphomevalonate decarboxylase; mvaK2, phosphomevalonate kinase; MVK, mevalonate kinase; HMGCR, hydroxymethylglutaryl-CoA reductase; HMGCS, hydroxymethylglutaryl-CoA synthase; ACAT, acetyl-CoA C-acetyltransferase; IDI, isopentenyl-diphosphate Delta-isomerase; SZDS, short-chain Z-isoprenyl diphosphate synthase; TPDDS, trans,polycis-decaprenyl diphosphate synthase; FDPS, farnesyl diphosphate synthase; ispS, isoprene synthase; cyc2, germacradienol/geosmin synthase; FPK, farnesyl phosphate kinase; NES1, (3S,6E)-nerolidol synthase; HVS, vetispiradiene synthase; CYP71D55, premnaspirodiene oxygenase; FNTB, protein farnesyltransferase subunit beta; STE24, STE24 endopeptidase; PCME, prenylcysteine alpha-carboxyl methylesterase; PCYOX1, prenylcysteine oxidase/farnesylcysteine lyase; FLDH, NAD+-dependent farnesol dehydrogenase; FOLK, 2-amino-4-hydroxy-6-hydroxymethyldihydropteridine diphosphokinase; FDFT1, farnesyl-diphosphate farnesyltransferase; SQLE, squalene monooxygenase; CAS1, cycloartenol synthase; ERG24, Delta14-sterol reductase; EBP, cholestenol Delta-isomeras; 24MCT, 24-methylenesterol C-methyltransferase ; SMO2, plant 4alpha-monomethylsterol monooxygenase ; 3BETAHSDD, plant 3beta-hydroxysteroid-4alpha-carboxylate 3-dehydrogenase; 3-KSR, 3-keto-steroid reductase ; ERG3, delta7-sterol 5-desaturase; DWF5, 7-dehydrocholesterol reductase; DWF1, Delta24-sterol reductase; CYP710A, sterol 22-desaturase; LSS, lanosterol synthase; CYP51, sterol 14alpha-demethylase; ERG25, methylsterol monooxygenase; CYP24A1, vitamin D3 24-hydroxylase; GPS, eranyl diphosphate synthase; GGPS, geranylgeranyl diphosphate synthase; TPS-Cin, 1,8-cineole synthase; HST, homogentisate solanesyltransferase; chiP, chemotaxis inhibitory protein; HPT, homogentisate phytyltransferase / homogentisate geranylgeranyltransferase; VTE3, MPBQ/MSBQ methyltransferase; VTE1, tocopherol cyclase; TOM, tocopherol O-methyltransferase; ent-CPS, ent-copalyl diphosphate synthase; KS, ent-kaurene synthase ; CYP701, ent-kaurene oxidase; KAO, ent-kaurenoic acid monooxygenase; GA20ox, gibberellin-44 dioxygenase; GA2ox, gibberellin 2beta-dioxygenase; GA3ox, gibberellin 3beta-dioxygenase.

Table S1 The full name of differentially expressed genes/proteins in Figure.4

| Abbreviations for differentially expressed genes/proteins | The full name of differentially expressed genes/proteins |
| --- | --- |
| PAL | phenylalanine ammonia-lyase |
| 4CL | 4-coumarate-CoA ligase |
| TC4M | trans-cinnamate 4-monooxygenase |
| HCT | shikimate-O-hydroxycinnamoyl transferase |
| C3H | coumaric acid 3-hydroxylase |
| CPR | NADPH-dependent cytochrome P450 reductase |
| COMT | caffeic acid 3-O-methyltransferase |
| F5H | ferulate-5-hydroxylase |
| CCoAOMT | caffeoyl-CoA O-methyltransferase |
| CCR | cinnamoyl-CoA reductase |
| CAD | cinnamyl-alcohol dehydrogenase |
| POD | peroxidase |
| LAC | laccase |
| DIR | dirigent |
| PLR | pinoresinol-lariciresinol reductases |
| SDH | secoisolariciresinol dehydrogenase |
| STS | stilbene synthase gene |
| CHS | chalcone synthase |
| CHI | chalcone isomerase |
| F3H | flavanone 3-hydroxylase |
| FNS | flavone synthase II |
| PPO | polyphenoloxidase |
| HCCR | hydroxycinnamoyl-CoA reductase |
| PGT1 | phlorizin synthase |
| FLS | flavonol synthase |
| F3M | flavonoid 3'-monooxygenase |
| BZ1 | anthocyanidin 3-O-glucosyltransferase |
| 3AT | anthocyanidin 3-O-glucoside 6''-O-acyltransferase |
| UGT78D1 | flavonol-3-O-rhamnosyltransferase |
| UGT73C6 | flavonol-3-O-L-rhamnoside-7-O-glucosyltransferase |

| Table S2 qPCR primers for detecting the expression of pathogenicity related genes in *Fusarium oxysporum* | |
| --- | --- |
| Primer’s name | Primer’s sequence |
| *FoFmk1* | Forward: CCAACCTCCTCCTCAACGC |
|  | Reverse: CCGACCACACATCAATAGCC |
| *FoXlnR* | Forward: CGAACCCACAATACCCTTACTG |
|  | Reverse: TGATGGCTTTGCCCGAG |
| *FoGas1* | Forward: CTTTGTCCCGTGGTTGGTT |
|  | Reverse: GCGGCTGTGTTGTTGTAGTG |
| *FoPl1* | Forward: AACTGGTGAGAAGGATGCTATGT |
|  | Reverse: CTGGTCTGCTTGAGGGTGAT |
| *FoFgb1* | Forward: TAACACATACCCCTACCGACG |
|  | Reverse: CCACCCATTACTAACAGCCC |
| *FoGBP* | Forward: ATCACATCTTGGTTTGG |
|  | Reverse: GAACCGCCTACACGGTGA |
| *FoActin* | Forward: GAGACATTCAACGCCCCAG |
|  | Reverse: GGTAAGATCACGACCAGCCA |

The cDNA sequences of *L. regale* and ‘Siberia’ ethylene-responsive transcription factor 4

>*LrERF4*

ATGGCTGCAATCTCGGACGATAACTCCGCTCTAGAACTCATCCGAGAGCACCTCTTCGGCGACAACCTCGCCGCCCCTCTCGACGCCTTCCTCGCCACCCCACCTCCCTGTAATTTCCACCACCTCACTCCATTCCACTCTATCCCTAGTCATCACTTTGTTCCAATCAACGACGATGTCACCGTGTCAGTCTCTGACTTCCTTGACCTCACAGCCTGCTACGAGCCTGAACCGGCCGTACCCATGATCCGGTTCGGCTCCACTGGCCGCAGCCCGTCGCTCTCTCTTGCTGTCTCACTGCCTGCTCCCCCAAAAGTAGAGTGGGCAGAGCCCACACTCCACGGTCCGCCGGCCGCAGCAGCCTGCGAAAGCCGGCGGTACCGTGGAGTGCGGCAGCGGCCGTGGGGGAAGTATGCAGCCGAGATCCGCGATCCCAACCGCAGGGGTTCACGAGTCTGGCTGGGTACTTTCGACACAGCAGTCGAGGCTGCACGAGCCTACGACCGTGCAGCTTTCGACCTGCGCGGCCGCAAAGCGATCCTGAACTTTCCTAATGAGGTCAGAATCCCTGAGGACCGTGCGGTACCGCAGCCGCAGATAGCGGCTGCTTCGGGAAAGAAGAGGTCGAGGCAATCGACGGAGGTGGAGGTTGAAATGAGGCCGATTAAGAGGGAGAGGTCACCGGAGACAGAGAGCGAGCCGGAGGTTGAAATGCTGAGCATGGCGAGCTGGATGGGGGTGTGGGATGGGGGAGACGTTAGTGGTAGCTTCGAGTTGCCGCCGCTGTCCCCGATGTCGCCTCATCCACAGATGAGGTATCTGGTTGTATCCTGA

>‘Siberia’ *ERF4*

ATGGCTGCAATCTCGGACGACAACTCCGCTCTAGAACTCATCCGAGAGCACCTCTTCGGCGACAACCTCGCCGCCCCTCTCGACACCTTCCTCGCCGCCCCACCTCCCTCTAATTTCCACCACTTCACTCCATTCCACTCTATCTCCGGTCACCACTTTGTTCCAATCAACGACGATGTCACTGTCTCAGTCTCTGACTTCCTTGACCTCACAGCCTGCTACGATCCTGAACCGGCCGTACCCATGATCCGGTTCGGCTCAACTGGCAGAAGTTCGTCGCTCTCTCTTGCTATATCTCTGCCCACTCCCCCGAAGGTAGAGTGGACAGAGCCCCTACTTCACGGCCCGCCGACCGCAGCAGCGTGCGACGGCCGGCGGTACCGTGGAGTGCGGCAGCGGCCGTGGGGGAAGTATGCGGCCGAGATCCGCGACCCCAACCGCAGGGGTTCACGGGTCTGGCTAGGTACTTTCGACACGGCAGTCGAGGCTGCCCGAGCCTATGATCGTGCAGCTTTCGACCTGCGCGGCCGCAAAGCGATCTTAAACTTTCCTAATGAGGTCAGAATCACCGAGGACCGAGCGGTACCGCAGCCGCAGATAGCGACTGCTTCGGGGAAGAAGAGGTCTAGGCAATCGTCGGAGGTGGAGATTGAAATGAGGCCGGTTAAGAGGGAGAGGTCACCGGAGATAGAGAGCGAGCCGGAGATTGAAATGCTGAGCATGGCGAGCTGGATGGGGGTTTGGGATGGGGGAGACATTAGTGGTAGCTTCGAGTTGCCACCGCTATCCCCGATGTCGCCTCATCCACAGATGAGGTATCTGGTTGTATCCTGA

| Table S3 qPCR primers for detecting the expression of phenylpropanoid biosynthesis related genes in *L. regale* | |
| --- | --- |
| Primer’s name | Primer’s sequence |
| *ERF4* | Forward: AAATGAGGCCGATTAAGAGGGAG |
|  | Reverse: GAGGCGACATCGGGGACAG |
| *PAL* | Forward: GATTGTTTGAGGCGGAGTTGAA |
|  | Reverse: CATTCCACTCCTTCAAGCACTCA |
| *4CL* | Forward: GCACACCGGAGATGTTGGTCTA |
|  | Reverse: TTTTACCTGTTTGTGGACAAAGCG |
| *F5H* | Forward: CTAATCAATAAACCTATCCAACGCCA |
|  | Reverse: AGGCTGAGAACCCGCTGGAT |
| *CAD* | Forward: AGCCTGGTAAATCACTTGGTGTCAT |
|  | Reverse: TTTGCTGCGGGTCAGACGAT |
| *COMT* | Forward: GGTTGAGGCTGGGGTTAATTTTG |
|  | Reverse: CAGGGCGTCGTAGCAGTTCTTC |
| *CCoAOMT* | Forward: ATGATAACACTCTTTGGTTTGGTAGCG |
|  | Reverse: GAAACACGCCTACAAATGGTGAGTC |
| *LAC* | Forward: CAACAACACACCAGTAGCACACCC |
|  | Reverse: ATTCGTAAGAAACTGGGTCAGGAGC |
| *CYP* | Forward: CGAAAGGTTTGCCCACGGTAG |
|  | Reverse: GGATTAAATTACCCCCTTCTGCCAT |
| *CHS* | Forward: AAGAGAAGTTACGCACAACAAGGCA |
|  | Reverse: TGGTATGGGGAGGCTACGGAGT |
| *CHI* | Forward: CACAGAAGCTGAAGGGGTTGC |
|  | Reverse: GGAAATGCGGAAGGCCAAG |
| *F3M* | Forward: TGGATGTGAAGGGAAGCGACT |
|  | Reverse: CCCATACGCCTGCTTCGC |
| *F3H* | Forward: ACCACCAGGCGGTAGTGAACT |
|  | Reverse: GGCTTCTTCACAACCTCCTGC |
| *FLS* | Forward: TATCCCAAATGCTCTTGTTATCCAC |
|  | Reverse: TTTTGCAGGATTCTCATCACTAACA |

Table S4 qPCR primers for detecting the expression of phenylpropanoid biosynthesis related genes in lily 'Siberia'

| Primer’s name | Primer’s sequence |
| --- | --- |
| *ERF4* | Forward: GTCGGAGGTGGAGATTGAAATG |
|  | Reverse: ACAACCAGATACCTCATCTGTGGAT |
| *PAL* | Forward: GAGTTGTTGCCCGAGTCTGATTTG |
|  | Reverse: CGGTCCAGCCTTCCTCAAACTT |
| *4CL* | Forward: ATTCAGAACAAGGTCGCCAAGC |
|  | Reverse: CAGCAGCAGCCAACTCCTTCTT |
| *F5H* | Forward: CATGGGGTACTTGCATATGGTCA |
|  | Reverse: GTAACTTATGGCTATGTTGGCTGGT |
| *CAD* | Forward: GTGTTGCTGTTGACGGCCGATG |
|  | Reverse: CTTGTTGCCGTTGCCCACCAG |
| *CCoAOMT* | Forward: ATTGGCTATGACAACACCCTATGGA |
|  | Reverse: GATTCTGGGATCAGCAGCCAACGCC |
| *CCR* | Forward: AAGCTTCAAGGGTGTGATCGATTA |
|  | Reverse: TCGGGAACCCAACGACCTTA |
| *CHS* | Forward: CACAGCAAGAAGCACGTACAGCAC |
|  | Reverse: AAGCAGCAAAGGATAGAGGCACG |
| *CHI* | Forward: GACAAGCGTGGTTACCCAGAGC |
|  | Reverse: AGAAGACGAGGGGAGTCCGAAC |
| *F3M* | Forward: GATGGTGACGTTCATGACAGCGA |
|  | Reverse: GTTTCTCATTCTTCCCATACGCCTG |
| *F3H* | Forward: TTATTACACTACCCACCTCAAACAG |
|  | Reverse: CCATCTTGAAAGTTGATCTGCA |
