# Supplementary figures and images for: Integrated multi-omics investigation revealed the importance of phenylpropanoid metabolism in the defense response of Lilium regale Wilson to fusarium wilt
Source: Hortic Res. 2024 May 20;11(7):uhae140. doi: 10.1093/hr/uhae140 (PMC11233880; doi:10.1093/hr/uhae140)

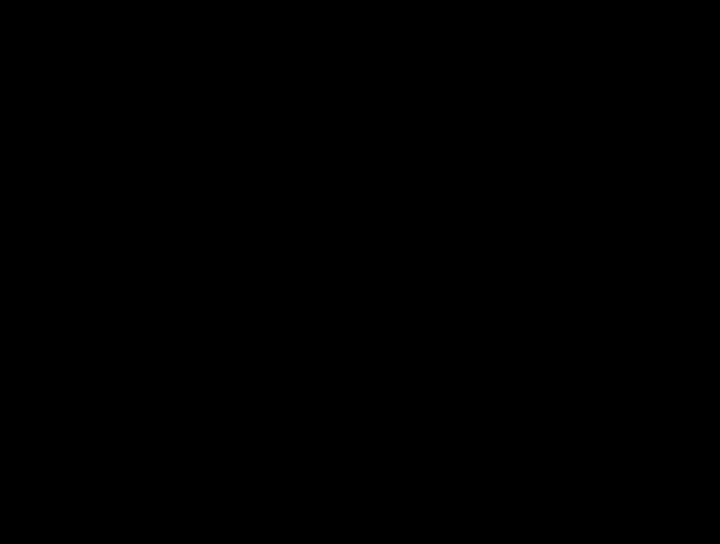

Supplement: Web_Material_uhae140 [file web_material_uhae140.zip › Dynamic fig 1.gif]

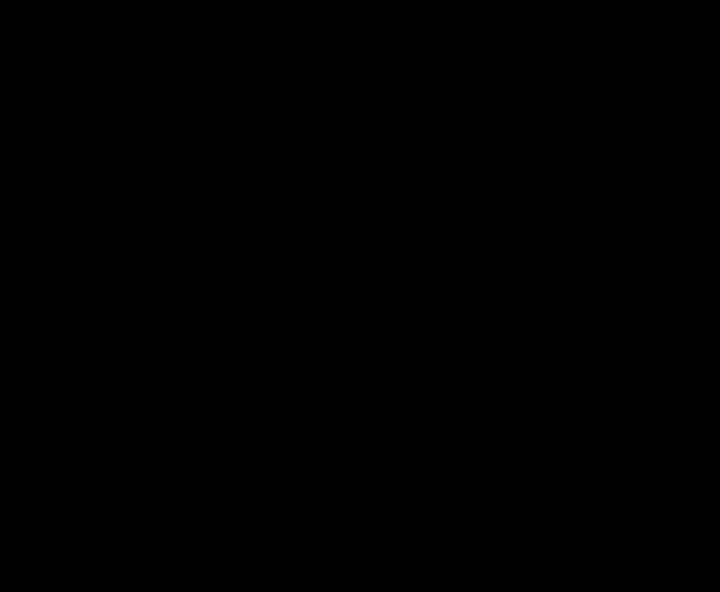

Supplement: Web_Material_uhae140 [file web_material_uhae140.zip › Dynamic fig 2.gif]

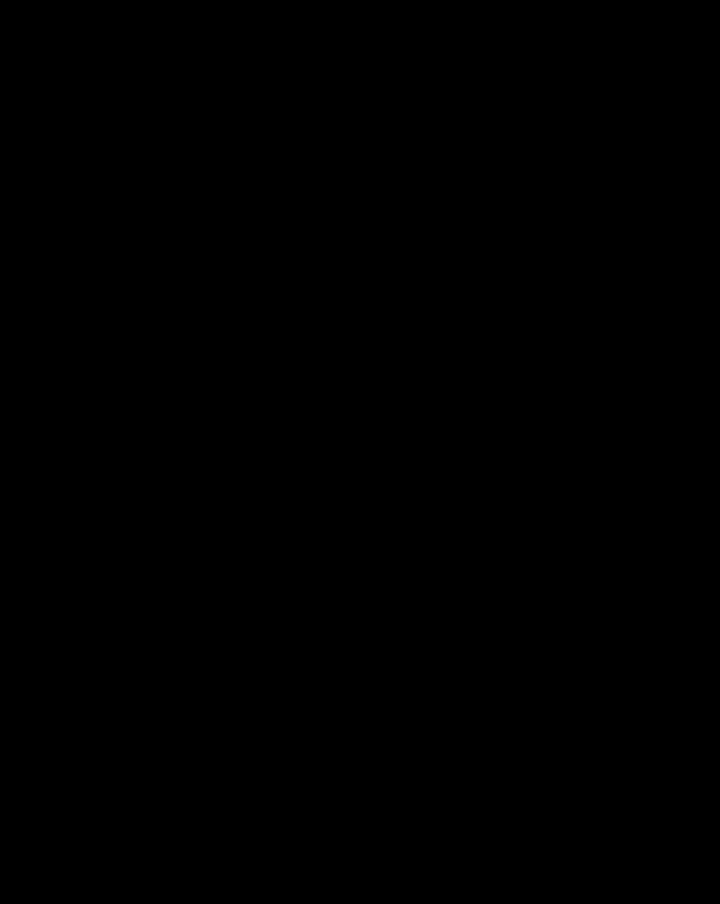

Supplement: Web_Material_uhae140 [file web_material_uhae140.zip › Dynamic fig 3.gif]

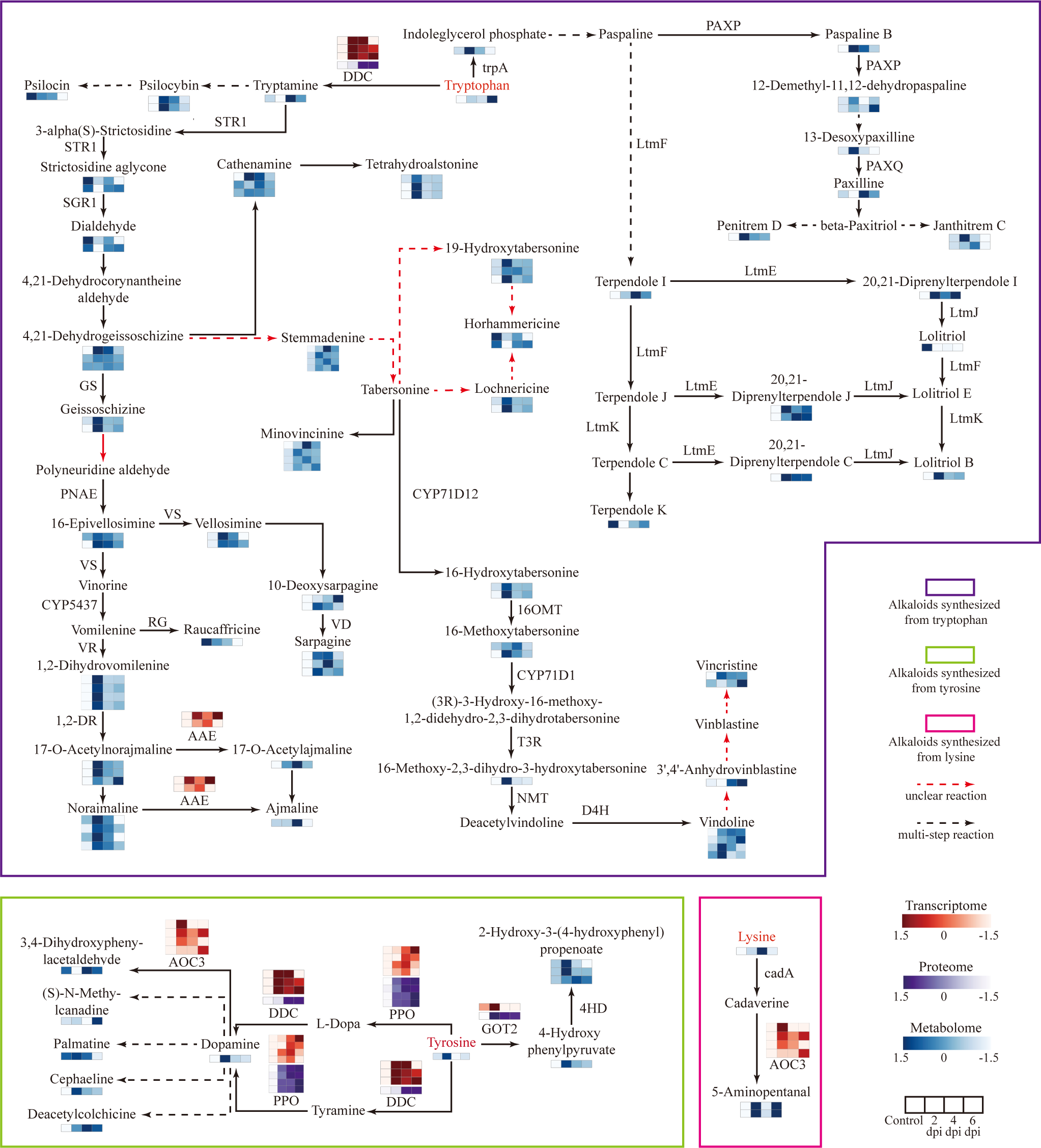

Supplement: Web_Material_uhae140 [file web_material_uhae140.zip › Fig S1.tif]

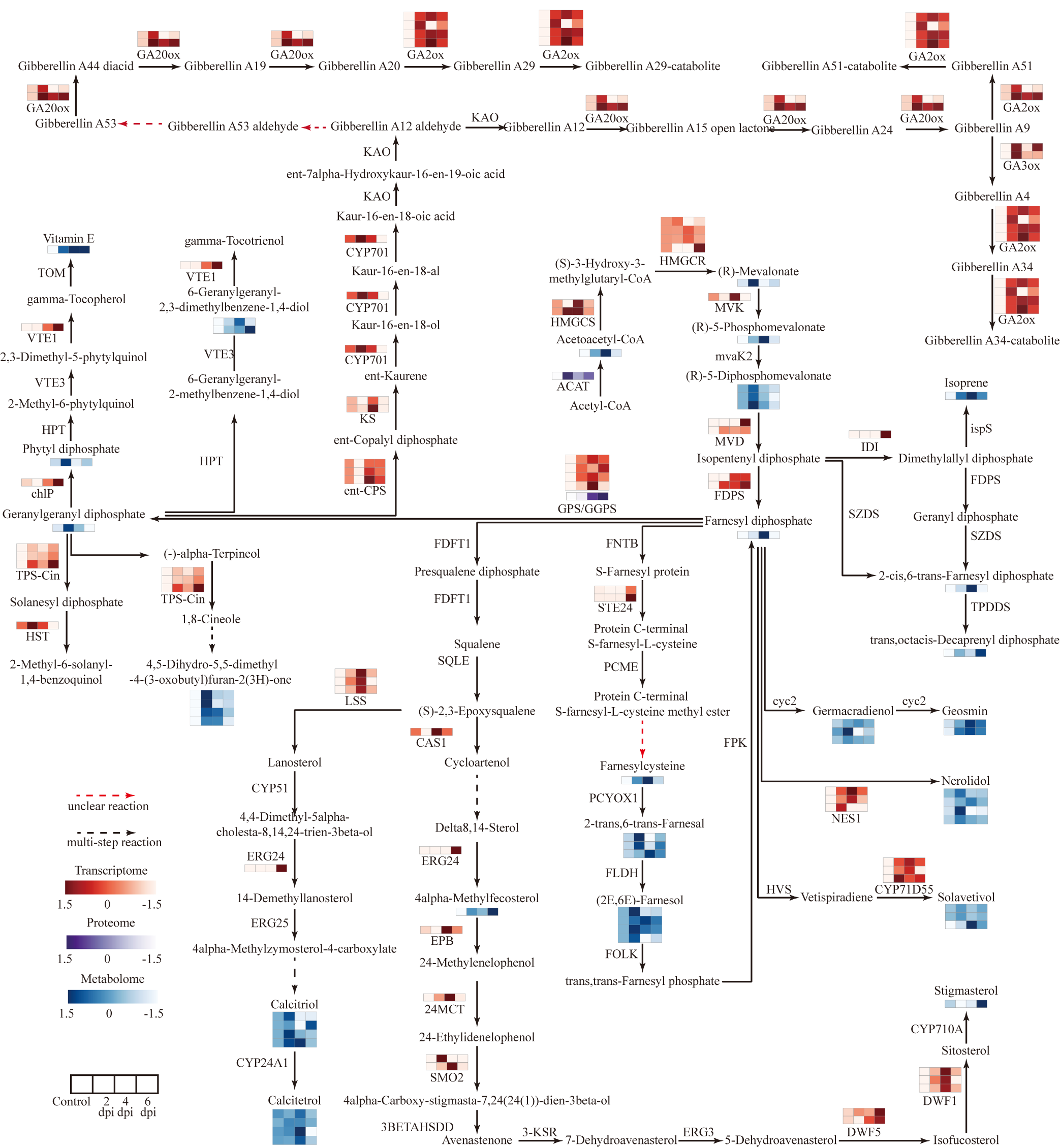

Supplement: Web_Material_uhae140 [file web_material_uhae140.zip › Fig S2.tif]
